# TGFβ induces an atypical EMT to evade immune mechanosurveillance in lung adenocarcinoma dormant metastasis

**DOI:** 10.1038/s43018-025-01094-y

**Published:** 2026-01-05

**Authors:** Zhenghan Wang, Yassmin Elbanna, Inês Godet, Siting Gan, Lila Peters, George Lampe, Yanyan Chen, Joao Xavier, Morgan Huse, Joan Massagué

**Affiliations:** 1 Cancer Biology and Genetics Program, Sloan Kettering Institute, Memorial Sloan Kettering Cancer Center, New York, NY USA; 2 Immunology Program, Sloan Kettering Institute, Memorial Sloan Kettering Cancer Center, New York, NY USA; 3 Gerstner Sloan Kettering Graduate School, Memorial Sloan Kettering Cancer Center, New York, NY USA; 4 Computational and Systems Biology Program, Memorial Sloan Kettering Cancer Center, New York, NY USA; 5 The Alan and Sandra Gerry Metastasis and Tumor Ecosystems Center, Memorial Sloan Kettering Cancer Center, New York, NY USA; 6 Present Address: Key Laboratory of Multi-Cell Systems, Shanghai Institute of Biochemistry and Cell Biology, Center for Excellence in Molecular Cell Science, Chinese Academy of Sciences, Shanghai, China; 7 Present Address: Department of Biochemistry and Molecular Biophysics, Columbia University Irving Medical Center, New York, NY USA; 8 Present Address: Specialized Microscopy Shared Resource, Herbert Irving Comprehensive Cancer Center, Columbia University Irving Medical Center, New York, NY USA

**Keywords:** Metastasis, Non-small-cell lung cancer, Cancer

## Abstract

Different forms of epithelial-to-mesenchymal transition (EMT) manifest during tumor progression. Little is known about the mechanistic basis and functional role of these distinct EMTs. We explored this question in lung adenocarcinoma (LUAD) primitive progenitors, which are competent to enter dormancy in response to transforming growth factor-β (TGFβ) upon metastatic dissemination. The TGFβ response in these cells includes growth arrest and a full EMT that subsequently transitions into an atypical mesenchymal state of round morphology and lacking actin stress fibers. TGFβ drives this transition by inducing expression of the actin depolymerizing protein gelsolin, which converts a migratory, stress-fiber-rich phenotype into a cortical actin-rich, spheroidal state. This transition lowers the biomechanical stiffness of metastatic progenitors and protects them from killing by cytotoxic lymphocytes. Gelsolin-deficient LUAD progenitors can enter dormancy but succumb to immune surveillance. Thus, quiescent LUAD metastatic progenitors undergo an atypical EMT to avert immune surveillance during TGFβ-driven metastatic dormancy.

## Abstract (web summary)

Wang et al. report that LUAD cells entering dormancy transition into a TGFβ-induced atypical mesenchymal state characterized by a soft spheroidal morphology and strong EMT signature, which protects disseminated cancer cells from immune clearance.

## Main

Cancer cells that disseminate from a tumor to distant sites enter a period of dormancy that may persist from months to decades before giving rise to detectable metastasis. Adjuvant therapy treatments seek to prevent overt metastasis by eliminating residual malignant cells during this dormancy period. Efforts to improve adjuvant therapy are hindered by an insufficient understanding of the molecular mechanisms that preserve the long-term viability of dormant metastatic cells. Identifying these mechanisms is necessary to improve treatments and prevent relapse^1–3^.

EMTs are manifestations of phenotypic plasticity that have important roles in development, injury repair, cancer invasion and metastasis^4–6^. During an archetypical EMT, epithelial cells lose apicobasal polarity, remodel cell adhesion contacts, assemble contractile actin stress fibers, adopt a spindly morphology, gain anteroposterior polarity and become motile. EMTs are driven by specific transcription factors, and in carcinoma cells, are closely associated with migratory and invasive behavior contributing to tumor dissemination and metastasis^7,8^. Cancer cells also present states characterized by an incomplete expression of EMT markers^9^ or adopt amoeboid features as part of the EMT phenotypic spectrum^10^. Despite the current consensus that cancer cells can adopt a spectrum of EMT states, it remains unclear what determines the extent and purpose of an EMT response during the different phases of metastasis, particularly the dormancy phase.

TGFβ is a major inducer of EMT responses in normal and malignant cells^5,11^ and is also an inducer of metastatic dormancy^12–14^. Disseminated carcinoma cells with competence to seed dormant metastasis express developmentally primitive progenitor markers, a capacity to enter quiescence and the ability to elude immune surveillance^15–18^. In experimental models, dormant metastatic cells predominantly reside in perivascular niches^19,20^, where TGFβ, secreted from diverse sources within the microenvironment, induces the entry of these cells into proliferative quiescence^12–14^. In this state, carcinoma cells are thought to evade immune surveillance by downregulating the expression of major histocompatibility complex class I (MHC-I) molecules^21–24^, natural killer (NK) cell receptor ligands^15^ and the STING pathway^18^. Re-entry of dormant metastatic cells into the cell cycle reactivates the expression of these immune activators, leading to cancer cell elimination by cytotoxic T lymphocytes (CTLs) and NK cells^15,18^. These insights suggest that metastatic dormancy is established by malignant progenitors fluctuating between an immune-evasive, slow-cycling state and a proliferative state that undergoes immune-mediated elimination^3,25,26^. Importantly, TGFβ has a critical role in enabling both the immune-evasive dormant state in primitive metastatic progenitors and an invasive phenotype in proliferative carcinoma cells^11^, raising questions about the nature of the EMT response in each of these states.

CTLs and NK cells kill cancer cells by forming an immunological synapse that mediates the delivery of perforin, granzymes and other pro-apoptotic factors to the target cell^27–29^. Synapse formation depends on the complement of activating ligands on the surface of target cells but is also affected by the biomechanical properties of these cells^30–33^. Increasing the stiffness of target cells elicits stronger cytotoxic responses, a process termed immune mechanosurveillance^33,34^. In large tumors, TGFβ acts as a direct suppressor of immune effector cells, but during dormancy, solitary disseminated cells persist in the presence of immune surveillance. In this context, the spindly architecture induced by TGFβ-dependent EMT would be expected to increase cell stiffness, thereby sensitizing metastatic cells to killing by CTLs. How metastatic cells evade this fate has remained unclear.

Given the essential roles of TGFβ in metastasis initiation, dormancy and progression^11^, we investigated the EMT state of dormant metastatic cells. Focusing on LUAD, our work reveals that dormant metastatic progenitor cells undergo a striking morphological transition as they enter TGFβ-induced dormancy after extravasation. In contrast to the stress-fiber-rich, fibrogenic EMT response induced by TGFβ in overtly metastatic cells^35,36^, we show that dormancy-competent LUAD progenitors respond to TGFβ with cell cycle arrest and an atypical EMT state that is devoid of actin stress fibers. We identify TGFβ-dependent expression of the actin depolymerizing protein gelsolin as a mediator of this transition. The resulting decrease in cell stiffness avoids immune mechanosurveillance and enhances survival during long-term metastatic dormancy before overt metastases.

## Results

### A morphological transition in dormant LUAD cells

**Fig. 1:**
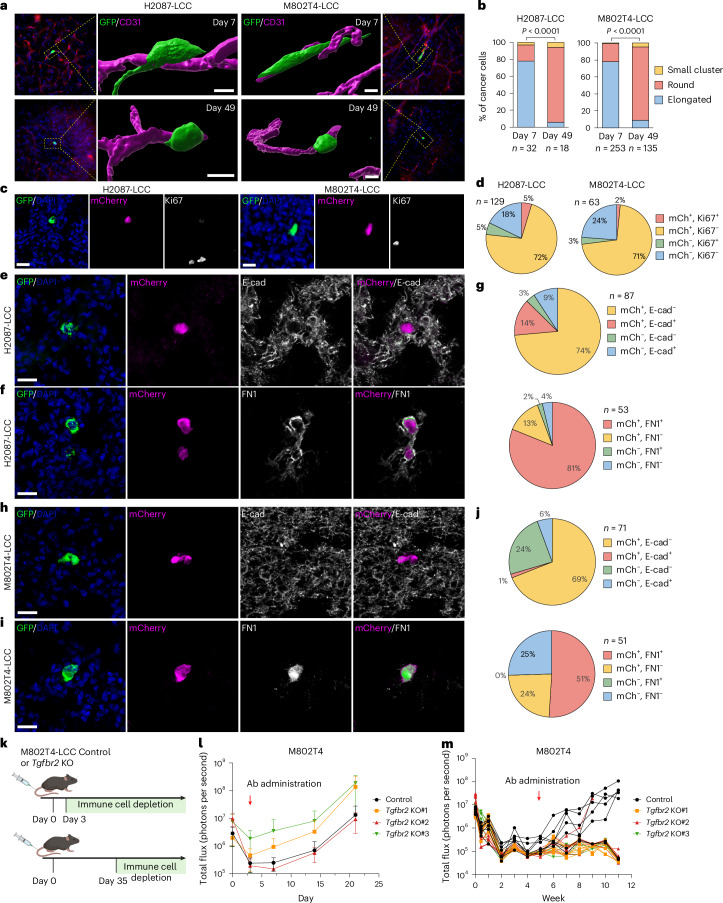
Vital TGFβ signaling in dormant LUAD cells. **a**, Three-dimensional reconstructed images of H2087-LCC and M802T4-LCC cells disseminated in brain parenchyma at the indicated time point after intracardiac inoculation into athymic mice and B6(Cg)-*Tyr*^*c-2J*^/J (B6-albino) mice, respectively. Cancer cells were visualized by GFP immunofluorescence (IF) (green) and brain capillaries by CD31 IF (magenta). Scale bar, 10 μm. **b**, Quantification of elongated, spheroidal and microclustered cancer cells (fewer than ten cells in the same focal plane) in **a**. H2087-LCC cells: *n* = 32 lesions (day 7) and *n* = 18 lesions (day 49), from six mice per group. M802T4-LCC cells: *n* = 253 lesions (day 7) and *n* = 135 lesions (day 49), from five mice per group. Chi-squared test. **c**, Representative images of H2087-LCC and M802T4-LCC cells expressing a TGFβ reporter in the lungs of athymic mice and B6129SF1/J mice, respectively, 7 weeks after intravenous injection. Cancer cells were visualized by GFP IF (green), the TGFβ mCherry reporter activity (magenta), Ki67 proliferation marker (white) and DAPI (blue). Scale bar, 20 μm. **d**, Quantification of TGFβ mCherry reporter and Ki67 expression in H2087-LCC (*n* = 129 lesions from five mice) and M802T4-LCC (*n* = 63 lesions from five mice) cells in the lungs of athymic and B6129SF1/J mice, respectively, 7 weeks after intravenous injection. **e**,**f**, Representative IF images of H2087-LCC in the lungs of athymic mice 7 weeks after intravenous injection. GFP^+^ cancer cells (green), TGFβ mCherry reporter (magenta), E-cadherin or fibronectin (FN1) (white) and DAPI (blue). **g**, Quantification of TGFβ mCherry reporter and E-cadherin (*n* = 87 lesions from four mice) or FN1 (*n* = 53 lesions from four mice) expression in disseminated H2087-LCC cells in the lungs of athymic mice. **h**,**i**, Representative IF images of M802T4-LCC in the lungs of B6129SF1/J mice 7 weeks after intravenous injection. GFP^+^ cancer cells (green), TGFβ mCherry reporter (magenta), E-cadherin (white) and DAPI (blue). Scale bar, 20 μm. **j**, Quantification of TGFβ mCherry reporter and E-cadherin (*n* = 71 lesions from five mice) or FN1 (*n* = 51 lesions from five mice) expression in M802T4-LCC in the lungs of B6129SF1/J mice 7 weeks after intravenous injection. **k**–**m**, Schematic representation of the experimental design, created with Biorender.com (**k**), and tracking of wild-type and *Tgfbr2* knockout (KO) M802T4-LCC cells intravenously inoculated in B6129SF1/J mice, followed by antibody-mediated depletion of NK, CD4^+^ and CD8^+^ T cells from day 3 (**l**) or day 35 (**m**) after inoculation. *n* = 6 mice for *Tgfbr2* KO#3 group and *n* = 7 mice for the rest of the groups. Data in **l** are means; error bars, s.d. Source data

**Extended Data Fig. 1:**
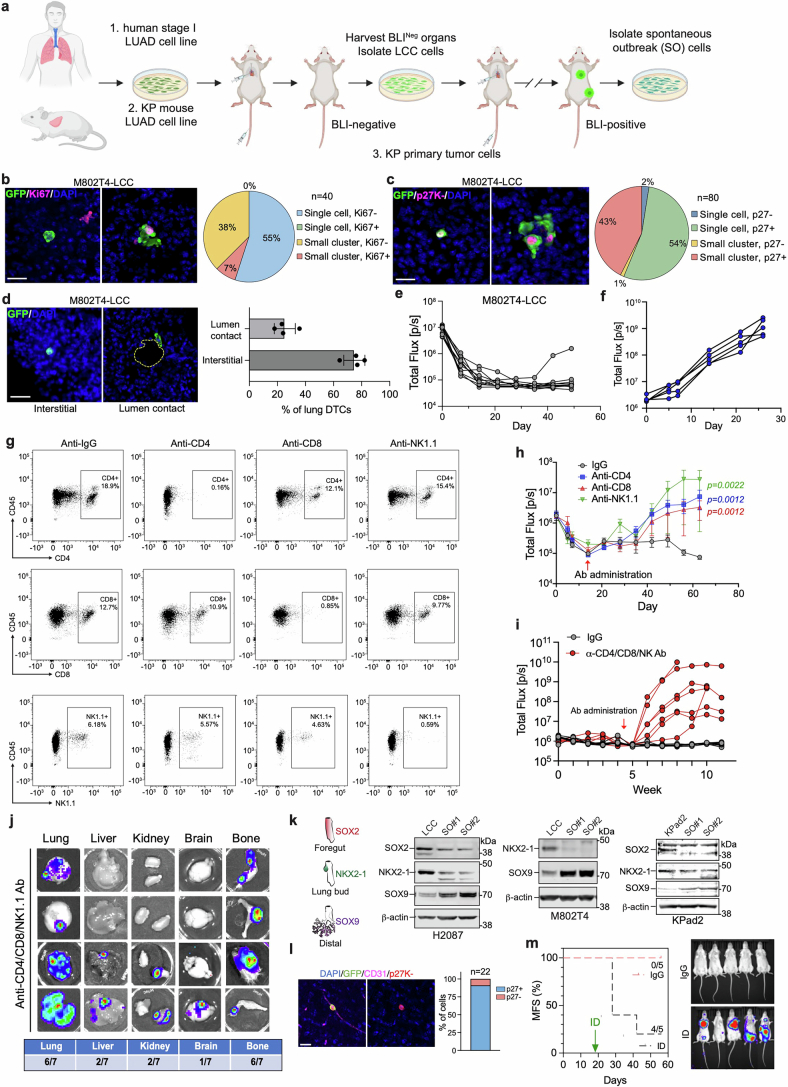
Generation and features of dormant LUAD metastasis models. **a**, Schematic protocol for the isolation of human or mouse LUAD cancer cells that are competent to enter dormancy and establish latent metastasis (created with Biorender.com). **b** and **c**, Representative IF images and quantification of proliferation status for single cell or small cluster ( < 10 cells in the same focal plane) of M802T4-LCC cells in the lungs of B6129SF1/J mice 7 weeks after intravenous injection. n = 40 and n = 80 lesions respectively, from 5 mice. The cells expressed green fluorescent protein (GFP), and firefly luciferase for bioluminescence imaging (BLI). Ki67 and mScarlet-p27K(-) reporter in magenta and DAPI in blue. Scale bar: 20 μm. **d**, Representative images and quantification of interstitial and lumen contact M802T4-LCC cells in the lung of B6129SF1/J mice 7 weeks after intravenous injection. n = 4 mice. Data are mean ± SD. Scale bar: 20 μm. **e**, BLI tracking the long-term persistence and metastatic outbreaks of M802T4-LCC cells after intravenous inoculation in the lungs of B6129SF1/J mice. 1 out of 10 mice developed overt metastases. **f**, BLI tracking of M802T4-LCC tumor growth after intravenous injection into NSG mice. n = 5 mice. **g**, Representative flow cytometry analysis of immune population in peripheral blood of B6129SF1/J mice injected with indicated antibody. **h**, BLI tracking of M802T4-LCC cells intravenously inoculated into B6129SF1/J mice followed by antibody-mediated depletion of the indicated immune cells. Data are mean ± SEM; n = 6 (IgG) or 7 mice per group; two-sided Mann-Whitney U-test. **i**, BLI tracking of M802T4-LCC cells intracardiacally inoculated into B6-Albino mice followed by antibody-mediated depletion of NK, CD4+ and CD8 + T cells. n = 7 mice per group. **j**, Representative ex vivo BLI images of indicated organs from mice upon immune depletion in **i**. Quantification of outbreaks in each organ shown at the bottom. **k**, Western immunoblots for lung development transcription factors (as shown in the schematic) in H2087-LCC cells, M802T4-LCC cells, KPad2 cells, and derivatives of these cells established from spontaneous outbreaks (SO). β-actin was used as loading control. **l**: Representative images and quantification of proliferation status of KPad2 cells in brain of B6-Albino mice 25 days after intracardiac injection. n = 22 lesions from 3 mice. Cancer cells (GFP) in green, CD31 in red, mScarlet-p27K(-) reporter in magenta and DAPI in blue. Scale bar: 20 μm. **m**, Metastasis-free survival curve and representative BLI images of B6-Albino mice injected with KPad2 cells following by IgG treatment or immune depletion (ID) of NK, CD4+ and CD8 + T cells. n = 5 mice per group. Source data

We previously established the H2087-LCC model of dormant LUAD metastasis by in vivo selection of a latency competent cancer (LCC) cell population derived from a stage I RAS-mutant human LUAD tumor (Extended Data Fig. 1a)^15^. When inoculated into the arterial circulation of *Foxn1*^*nu*^ athymic mice, H2087-LCC cells populate perivascular sites in lungs, brain and other organs, remaining for months as solitary cells or small clusters (fewer than ten cells in the same focal plane) and progressing to overt metastasis if the host mice are subjected to NK cell depletion^15^. To generate an analogous immunocompetent model, we applied the LCC selection protocol to 802T4, a cell line derived from a primary LUAD in the *Kras*^LSL-G12D/+^; *Trp53*^flox/flox^ (KP) mouse model^37^. The resulting cell population, M802T4-LCC, persisted for months as single cells and rare small clusters in lungs upon inoculation into immunocompetent B6129SF1/J mice. Single disseminated cells showed absence of the Ki67 proliferation marker and expression of the cell cycle quiescence reporter mScarlet-p27K^−^ (ref. ^38^) (Extended Data Fig. 1b–c). Single disseminated cells localized to interstitial and luminal sites in the lungs (Extended Data Fig. 1d) and in perivascular niches in the brain parenchyma (Fig. 1a). Although recipient mice harboring disseminated M802T4-LCC cells showed few spontaneous outbreaks (Extended Data Fig. 1e), M802T4-LCC cells formed aggressive metastases when inoculated into immunodeficient NOD scid gamma (NSG) mice (Extended Data Fig. 1f). Antibody-mediated depletion of NK cells, CD4^+^ or CD8^+^ T cells in immunocompetent hosts (Extended Data Fig. 1g) caused widespread metastasis in the lungs of mice inoculated through the tail vein (Extended Data Fig. 1h), and in liver, kidney and adrenal glands, brain and bones of mice inoculated intracardially (Extended Data Fig. 1i,j). These results validated M802T4-LCC as a model of LUAD metastatic dormancy under systemic immune surveillance.

H2087-LCC cells expressed the pluripotency transcription factor SOX2, which specifies primitive foregut during development^39^, and M802T4-LCC cells expressed the proximal progenitor transcription factor NKX2-1, which marks early-stage lung bud progenitors (Extended Data Fig. 1k)^40^. By contrast, cell populations derived from spontaneous metastatic outbreaks (SO) in mice (H2087-SO and M802T4-SO cell populations) expressed the late-stage lung progenitor transcription factor SOX9 predominantly over SOX2 or NKX2-1 (Extended Data Fig. 1k). This pattern recapitulated a continuum of developmental stages that we previously observed by single-cell transcriptomics in patient-derived LUAD metastasis samples^17^. As a complementary model, we also used LCC cells derived from early-stage LUAD lesions in KP mice (KPad2 cells), as previously described^18^. KPad2 cells remain in a dormant state and reside in perivascular niches in the brain when inoculated into C57BL/6-derived B6-albino mice intracardially (Extended Data Fig. 1l). Depletion of NK and T cells resulted in rapid outbreaks (Extended Data Fig. 1m). KPad2 cells express high levels of SOX2 and low levels of SOX9 compared with KPad2-SO cells from spontaneous outbreaks in mice (Extended Data Fig. 1k).

**Extended Data Fig. 2:**
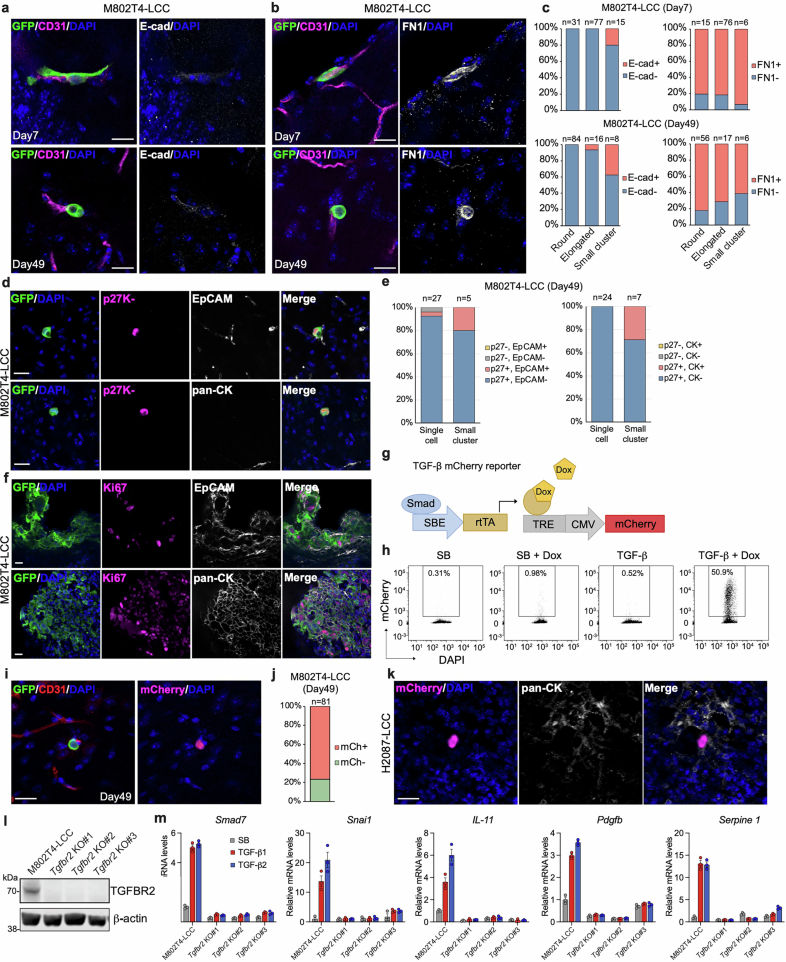
Mesenchymal markers and TGF-β signaling in disseminated LUAD cells. **a** and **b**, Representative IF images of M802T4-LCC cells in brain parenchyma at the indicated time point after intracardiac injection into B6-albino mice. GFP^+^ cancer cells (green), CD31^+^ blood capillaries (magenta), E-cadherin or FN1 (white), and DAPI (blue). Scale bar: 20 μm. **c**, Quantification of E-cadherin and FN1 IF in brain metastatic 802T4-LCC cells 1 week and 7 weeks after intracardiac inoculation. Number of lesions (from 5 mice) as indicated for each condition. **d** and **f**, Representative IF images of dormant M802T4-LCC cells or proliferative outbreaks in the brain of B6-Albino 7 weeks after injection. GFP^+^ cancer cells (green), Scarlet-p27K(-) or Ki67 (magenta), EpCAM or pan-cytokeratin (white), and DAPI (blue). Scale bar: 20 μm. **e**, Quantification of p27, EpCAM and pan-CK in brain metastatic M802T4-LCC cells 7 weeks after intracardiac inoculation. Number of lesions (from 4 mice) as indicated for each condition. **g**, Schematic representation of a doxycycline-inducible TGF-β/SMAD signaling reporter construct driving expression of mCherry. *SBE*, SMAD binding elements; *rtTA*, reverse tetracycline-controlled transactivator; *TRE*; Tet Response Element; *CMV*, cytomegalovirus promoter; *Dox*, doxycycline. **h**, Flow cytometry analysis of H2087-LCC cells expressing a TGF-β mCherry reporter under the indicated conditions. **i**, Representative IF images of M802T4-LCC cells expressing the TGF-β reporter in the brain of B6-albino mice 7 weeks after intracardiac injection. GFP^+^ cancer cells (green), mCherry (magenta), CD31^+^ blood capillaries (red), and DAPI (blue). Scale bar: 20 μm. **j**, Quantification of TGF-β mCherry reporter expression in M802T4-LCC cells (n = 81 lesions from 5 mice) in the brain of B6-albino mice 7 weeks after intracardiac injection. **k**, Representative IF images of H2087-LCC cells expressing the TGF-β reporter in the lung of athymic mice 7 weeks after intravenous injection. GFP^+^ cancer cells (green), mCherry (magenta), pan-cytokeratin (white), and DAPI (blue). Scale bar: 20 μm. **l**, Western immunoblot of TGFBR2 expression in wild-type and *Tgfbr2* KO M802T4-LCC cells. β-actin was used as loading control. **m**, qRT-PCR analysis of indicated gene expression in wild-type and *Tgfbr2* KO M802T4-LCC cells upon incubation with SB505124 (SB), TGF-β1 or TGF-β2 for 2 h. Data are mean ± SEM; n = 3 technical replicates. Representative results from two independent experiments are shown. Source data

To investigate the behavior of dormant H2087-LCC and M802T4-LCC cells in vivo, we analyzed these cells after dissemination to the brain, where the elongated nature of blood capillaries facilitated the morphological analysis of these cells in perivascular niches, and in the lungs, where higher numbers of disseminated cancer cells allowed quantitative analysis. Carcinoma cells extravasate in the brain between 3 days and 7 days after inoculation^41,42^. At 7 days after inoculating H2087-LCC into athymic mice and M802T4-LCC into B6-albino mice, cancer cells in the brain were situated around blood capillaries and showed a predominantly elongated morphology (Fig. 1a,b), as previously observed with other, more aggressive models of metastasis^42,43^. This morphology was consistent with reports that circulating cancer cells undergo EMT for migration through endothelia during metastatic extravasation^44^. Notably, the H2087-LCC and M802T4-LCC cells subsequently adopted a spheroidal morphology as they settled into long-term dormancy (Fig. 1a,b). The disseminated H2087-LCC and M802T4-LCC cells showed certain mesenchymal traits, including high expression of the mesenchymal marker fibronectin (*FN1*) and low expression of the epithelial marker E-cadherin, in association with the elongated morphology early after dissemination, as well as with the spheroidal morphology that became prevalent weeks after dissemination (Extended Data Fig. 2a–c). Dormant disseminated M802T4-LCC cells did not express EpCAM or cytokeratin, whereas proliferative outbreaks stained positive for these epithelial markers (Extended Data Fig. 2d–f).

### Vital TGFβ signaling in dormant LUAD cells

TGFβ1 and TGFβ2 produced by the microenvironment are known to regulate the dormant state of disseminated carcinoma cells^25,26^. To determine whether dormant solitary LUAD cells persistently harbored TGFβ signaling activity, we engineered a doxycycline-dependent TGFβ mCherry reporter^18^ into H2087-LCC and M802T4-LCC cells (Extended Data Fig. 2g,h). Mice were inoculated with these cells and switched to doxycycline-containing chow to allow TGFβ reporter expression. Long-term disseminated H2087-LCC and M802T4-LCC in the lungs (Fig. 1c,d) and brain (Extended Data Fig. 2i,j) showed persistent mCherry expression together with low expression of Ki67, E-cadherin and cytokeratin, and high expression of fibronectin (Fig. 1c–j and Extended Data Fig. 2k). These results indicate that dormant LUAD progenitor cells harbor sustained TGFβ signaling activity and mesenchymal traits during dormancy.

To evaluate the importance of TGFβ signaling for LUAD dormancy, we intravenously inoculated B6129SF1/J immunocompetent mice with wild-type M802T4-LCC cells or M802T4-LCC cells with knockout of the central TGFβ receptor subunit *Tgfbr2*, abolishing gene responses to TGFβ1 and TGFβ2 (ref. ^45^) (Extended Data Fig. 2l,m). We then subjected the mice to antibody-mediated depletion of NK cells, CD4^+^ T cells and CD8^+^ T cells either 3 days or 35 days after inoculation, to allow the metastatic outgrowth of disseminated cancer cells (Fig. 1k). This protocol provides a readout of the metastasis-initiating competence of latent cancer cell populations after specific perturbations^15,18^. Early depletion of NK and T cells led to the aggressive outgrowth of metastatic colonies in the lungs of all mice, and this growth was not inhibited by the knockout of *Tgfbr2* (Fig. 1l). By contrast, late depletion of NK and T cells led to metastatic outbreaks in five out of seven mice harboring disseminated M802T4-LCC cells, but in only one out of 21 mice harboring disseminated *Tgfbr2* knockout M802T4-LCC cells (Fisher’s exact test, *P* = 0.0012) (Fig. 1m). These results suggest that TGFβ is not essential for metastatic seeding of M802T4-LCC cells in the lungs but is important for the preservation of metastasis-initiating cells during prolonged dormancy.

### An EMT state lacking actin stress fibers

TGFβ is a potent inducer of EMTs in normal and malignant epithelial cells^11^. Aggressive metastatic cells derived from patients with LUAD and from KP mouse models respond to TGFβ by undergoing a typical EMT coupled with expression of a set of fibrogenic factors, which is critical for the ensuing metastatic outgrowth in the lungs^35^. Although the spindly morphology of H2087-LCC and M802T4-LCC cells upon extravasation (refer to Fig. a) was compatible with a classical EMT, the spheroidal configuration adopted by these cells during long-term dormancy was not. Mesenchymal traits have also been noted in dormant breast cancer cells^46–48^.

**Fig. 2:**
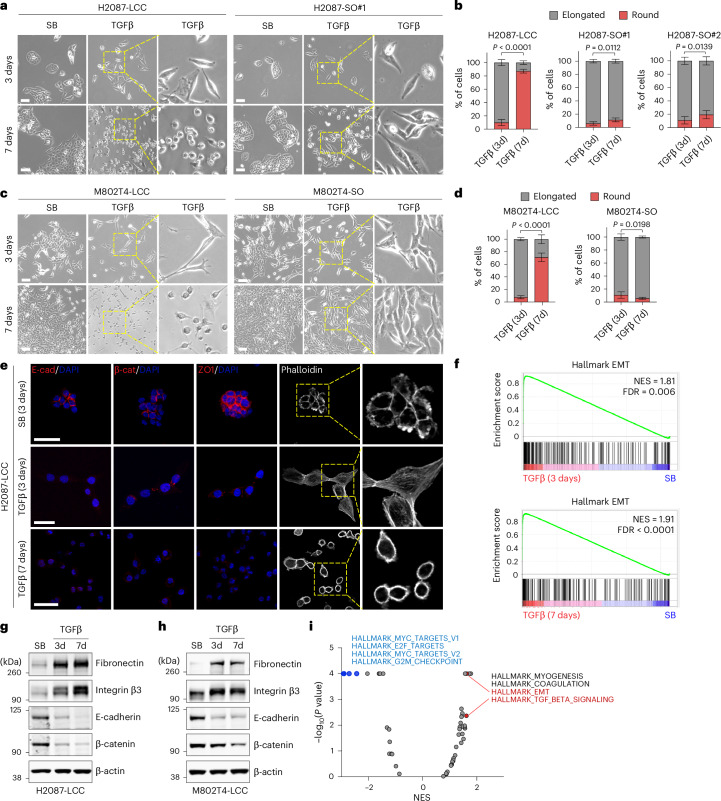
An EMT state lacking actin stress fibers. **a**,**c**, Representative bright-field images of indicated cell cultures after incubation with TGFβ for 3 days and 7 days. Scale bar, 200 μm. SB, SB-505124. **b**,**d**, Quantification of elongated and spheroidal cells upon TGFβ treatment for 3 days and 7 days. Cells from *n* = 10 images (LCC cells) or *n* = 8 images per condition (SO cells) were analyzed in **b**, and cells from *n* = 8 images per condition were analyzed in **d**. Data are means; error bars, s.d.; two-sided unpaired *t*-test. **e**, Representative IF images for the epithelial markers E-cadherin, β-catenin and zona occludens 1 (ZO-1), and of phalloidin staining in H2087-LCC cells incubated with TGFβ for 3 days or 7 days. Scale bar, 20 μm. **f**, Expression of a hallmark EMT gene signature in H2087-LCC cells after incubation with TGFβ for 3 days and 7 days versus SB control. NES, normalized enrichment score; FDR, false discovery rate. **g**,**h**, Western immunoblot analysis of the indicated proteins in H2087-LCC and M802T4-LCC cells after incubation with TGFβ for 3 days or 7 days, or with SB for 3 days. β-actin was used as a loading control. Representative results from two independent experiments are shown. **i**, Volcano plot of Hallmark gene signatures enriched in H2087-LCC cells incubated with TGFβ for 7 days versus 3 days. Source data

**Extended Data Fig. 3:**
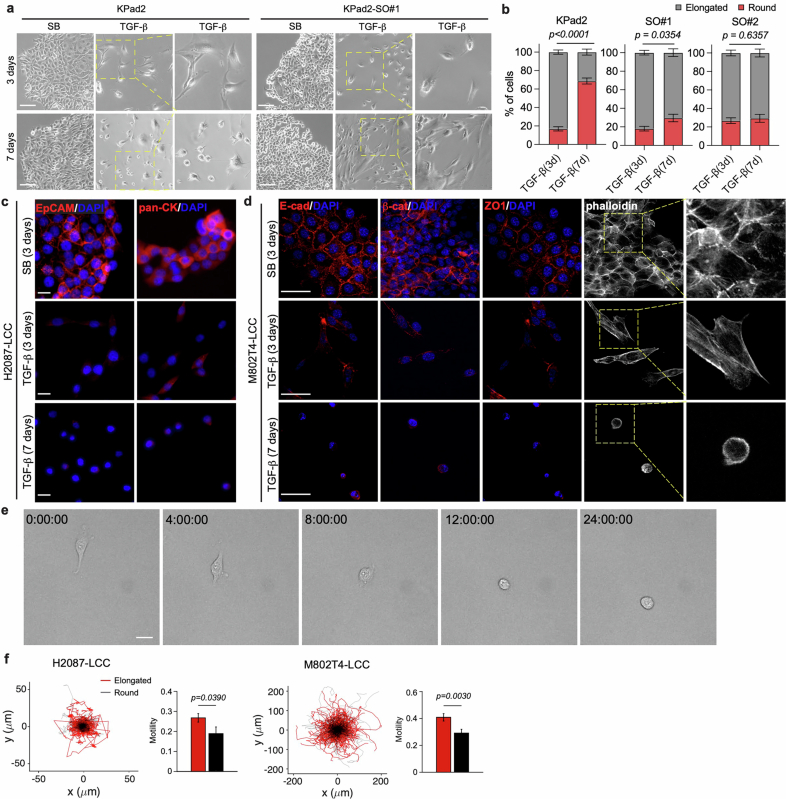
An EMT state lacking actin stress fibers. **a**, Representative bright-field images of KPad2 and SO cells cultures after incubation with TGF-β for 3 days and 7 days. Scale bar: 200 μm. **b**, Quantification of elongated and spheroidal cells upon TGF-β treatment for 3 days and 7 days (n = 6, 9, 6, 7, 6, 5 images, from left to right). Data are mean ± SD; two-sided unpaired *t*-test. **c**, Representative IF images of EpCAM and pan-cytokeratin staining in H2087-LCC cells incubated with TGF-β for 3 days or 7 days. Scale bar: 20 μm. **d**, Representative IF images for E-cadherin, β-catenin, and ZO-1, and of phalloidin staining in M802T4-LCC cells incubated with TGF-β for 3 days or 7 days. Scale bar: 20 μm. **e**, Representative still frames from time-lapse imaging of H2087-LCC cells incubated with TGF-β for 5 days and imaged for 24 h in the presence of TGF-β. Scale bar: 50 μm. **f**, Spider plots of migration tracks and quantification of motility in elongated LCC cells (n = 139 cells and n = 319 cells for H2087 and M802T4 respectively) after 3 days of incubation with TGF-β and spheroidal LCC cells (n = 64 cells and n = 282 cells for H2087 and M802T4 respectively) after 7 days of incubation with TGF-β. Data are mean ± SEM. Source data

To investigate the EMT response in dormancy-competent LUAD cells under sustained TGFβ stimulation, we incubated H2087-LCC, M802T4-LCC and KPad2 cells with TGFβ (100 pM TGFβ1, unless otherwise indicated) or the TGFβ receptor inhibitor SB-505124 (ref. ^49^), which sets a baseline by inhibiting endogenous TGFβ signaling in cell cultures^36^. The cells grew as epithelial clusters under normal culture conditions and responded to TGFβ by initially adopting a spindly morphology (Fig. 2a–d and Extended Data Fig. 3a,b), accompanied by a loss of E-cadherin, β-catenin, ZO-1, EpCAM and cytokeratin, and a gain of actin stress fibers (Fig. 2e and Extended Data Fig. 3c,d) and motility (Extended Data Fig. 3e and Supplementary Video 1), all hallmarks of a typical full EMT^6^. Notably, after 3 days of incubation with TGFβ, the LCC cells started to transition into a spheroidal morphology with an absence of stress fibers (Fig. 2a–d and Extended Data Fig. 3a,b) and decreased motility (Extended Data Fig. 3e,f and Supplementary Video 1), a phenotype that became prevalent after 7 days of incubation with TGFβ. The actin stress fibers appearing during the early phase of the TGFβ response were replaced with subcortical actin filaments by day 7, consistent with the observed transition from an elongated to a spheroidal morphology (Fig. 2e and Extended Data Fig. 3d). This transition resembled that of H2087-LCC and M802T4-LCC cells entering dormancy in vivo. By contrast, H2087-SO, M802T4-SO and KPad2-SO derivatives exhibited long-term EMT traits during prolonged incubation with TGFβ (Fig. 2a–d and Extended Data Fig. 3a,b), similar to those of aggressively metastatic LUAD cell lines^35^.

### Distinct EMT and quiescence responses in different LUAD progenitor states

**Fig. 3:**
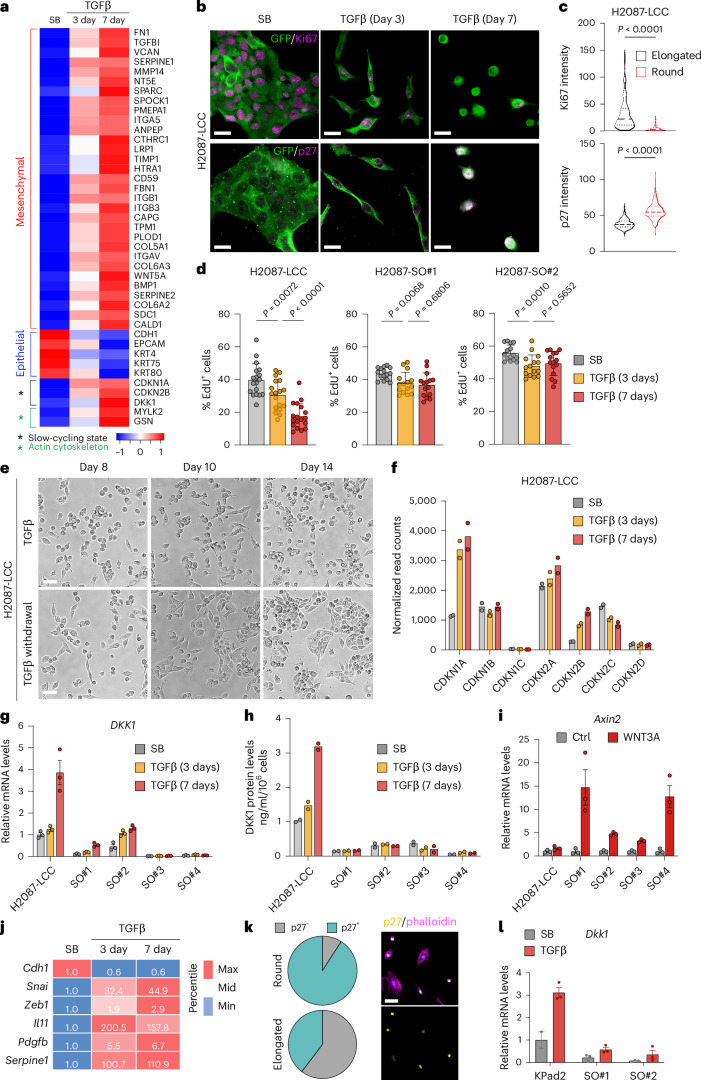
Quiescence-associated EMT responses to TGFβ. **a**, Heatmap showing the expression of differentially expressed EMT genes, key growth inhibitors and cytoskeleton regulators in H2087-LCC cells treated with SB, or TGFβ for 3 days or 7 days. **b**, Representative IF images of H2087-LCC cells after incubation with TGFβ for 3 days or 7 days. GFP^+^ cancer cells (green), Ki67 (magenta), p27KIP1 (magenta). Scale bar, 20 μm. **c**, Quantification of Ki67 and p27 IF intensity in elongated H2087-LCC cells after 3 days of incubation with TGFβ and spheroidal H2087-LCC cells after 7 days of incubation with TGFβ. *n* = 299 cells (elongated) and *n* = 116 cells (round) for Ki67 staining; *n* = 222 cells (elongated) and *n* = 265 cells (round) for p27 staining. Two-sided unpaired *t*-test. **d**, Quantification of EdU^+^ cells in H2087-LCC and SO cells upon TGFβ treatment for 3 days and 7 days. Results from *n* = 18 images (LCC cells) or *n* = 15 images per condition (SO cells) were analyzed. Data are means; error bars, s.d.; two-sided unpaired *t*-test. **e**, Representative bright-field images of H2087-LCC cell cultures pre-treated with TGFβ for 7 days and then switched to media containing TGFβ or SB for the indicated time periods. Scale bar, 200 μm. **f**, Normalized read counts of the indicated genes from RNA-seq results of H2087-LCC cells treated with SB, or TGFβ for 3 days or 7 days. *n* = 2 technical replicates. **g**, Quantitative PCR with reverse transcription (RT–qPCR) analysis of *DKK1* mRNA levels in H2087-LCC and SO cells treated with TGFβ for 3 days and 7 days. Data are means; error bars, s.e.m.; *n* = 3 technical replicates. **h**. DKK1 protein levels measured by ELISA in H2087-LCC and SO cells treated with TGFβ for 3 days and 7 days. Data are means; error bars, s.d.; *n* = 2 biological replicates. **i**, RT–qPCR analysis of *Axin2* mRNA levels in H2087-LCC and SO cells treated with recombinant human WNT3A for 2 h. Data are means; error bars, s.e.m.; *n* = 3 technical replicates. **j**, RT–qPCR analysis of EMT markers in KPad2 cells treated with SB, or TGFβ for 3 days or 7 days. Data are means; *n* = 3 technical replicates. **k**, Representative IF images of KPad2 cells treated with TGFβ for 7 days. Quantification of p27 staining in round and elongated KPad2 cells treated with TGFβ for 7 days. Scale bar, 50 μm. **l**, RT–qPCR analysis of *DKK1* mRNA levels in KPad2 and SO cells treated with TGFβ for 7 days. Data are means; error bars, s.e.m.; *n* = 3 technical replicates. All RT–qPCR results were from one representative experiment of two independent repeats. Source data

**Extended Data Fig. 4:**
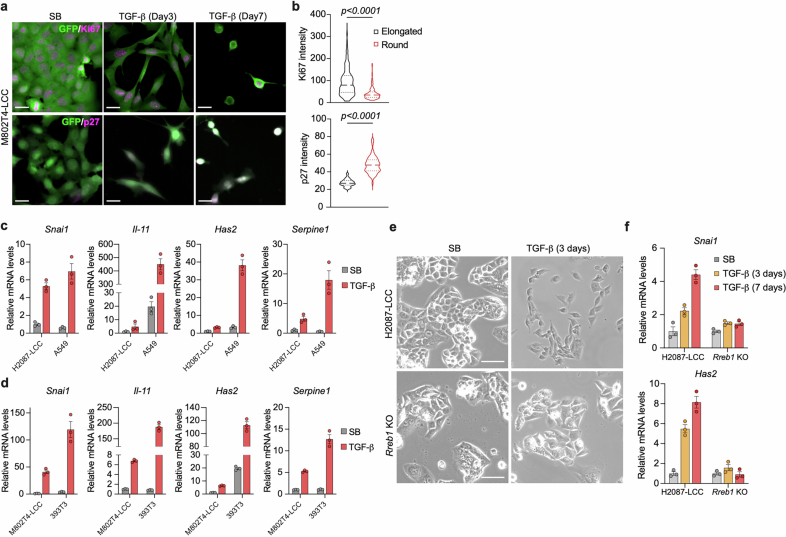
Distinct EMT responses in different LUAD progenitor states. **a**, Representative IF images of M802T4-LCC cells after incubation with TGF-β for 3 days or 7 days. GFP^+^ cancer cells (green), Ki67 (magenta) or p27KIP1 (magenta). Scale bar: 20 μm. **b**, Quantification of Ki67 and p27 IF intensity in elongated M802T4-LCC cells after 3 days of incubation with TGF-β and spheroidal M802T4-LCC cells after 7 days of incubation with TGF-β. Violin distributions are centered around the median (dashed line) with quartiles ranges displayed above and below (dotted lines). n = 513 cells (elongated) and n = 131 cells (round) for Ki67 staining, n = 121 cells (elongated) and n = 63 cells (round) for p27 staining. Two-sided unpaired *t*-test. **c** and **d**, qRT-PCR analysis of *Snai1* EMT-TF and three representative EMT-associated fibrogenic factors^35^ in the indicated cell lines upon TGF-β treatment for 2 h. Data are mean ± SEM; n = 3 technical replicates. **e**, Representative bright-field images of wild-type and *Rreb1* KO H2087-LCC cells incubated with TGF-β for 3 days. Scale bar: 200 μm. **f**, qRT-PCR analysis of *Snai1* and *Has2* expression in wild-type and *Rreb1* KO H2087-LCC cells incubated with TGF-β for 3 days or 7 days. Data are mean ± SEM; n = 3 technical replicates. All qRT-PCR results were from one representative experiment of two independent repeats. Source data

Despite the lack of stress fibers and motility, spheroidal H2087-LCC and M802T4-LCC cells continued to show an absence of epithelial markers (E-cadherin, β-catenin and ZO-1) after 7 days of incubation with TGFβ (Fig. 2e and Extended Data Fig. 3c,d). Gene set enrichment analysis of RNA sequencing (RNA-seq) data demonstrated the persistence of a strong EMT transcriptional program in H2087-LCC cells incubated with TGFβ for 7 days (Fig. 2f). Biochemical analysis confirmed an increase in fibronectin and integrin β3 (*ITGB3*) expression as mesenchymal state markers, and a downregulation of E-cadherin and β-catenin expression in H2087-LCC and M802T4-LCC cells incubated for 3 days or 7 days with TGFβ (Fig. 2g,h). EMT was among the top enriched signatures on day 7 compared to day 3 (Fig. 2i and Supplementary Table 1). We observed an upregulation of essentially all individual mesenchymal markers and downregulation of epithelial markers by day 7 compared to day 3 (Fig. 3a). Proliferation-related signatures were extensively downregulated by day 7 compared to day 3 (Fig. 2i). Entry of H2087-LCC cells into a slow-cycling state was confirmed by low expression of Ki67 and high expression of the quiescence marker p27KIP1 (p27)^50^ upon transitioning into the spheroidal phenotype in response to TGFβ (Fig. 3b,c and Extended Data Fig. 4a,b). H2087-SO cells showed a less pronounced growth-inhibitory response than H2087-LCC cells (Fig. 3d). H2087-LCC cells placed in regular media after incubating for 7 days with TGFβ transitioned back to an epithelial morphology, whereas cells that continued with TGFβ for up to 14 days retained a spheroidal morphology (Fig. 3e).

We have shown that TGFβ signaling blunts cell proliferation partly through the induction of CDK inhibitors^51,52^. TGFβ treatment for 3 days or 7 days increased the expression of *CDKN1A* and *CDKN2B* in H2087-LCC cells (Fig. 3f). Under growth-restrictive conditions, H2087-LCC cells additionally express the WNT inhibitor DKK1, which enforces dormancy in vivo^15^. In line with these findings, incubation with TGFβ for 7 days strongly increased the expression of DKK1 in H2087-LCC but not in H2087-SO cells (Fig. 3g,h). H2087-SO cell populations showed a diminished ability to resist WNT3A induction of the canonical WNT target gene *Axin2* compared to H2087-LCC cells (Fig. 3i). TGFβ also induced an EMT with a spindly state transitioning into a slow-cycling spheroidal state in KPad2 cells (Fig. 3j,k) and increased the expression of DKK1 in KPad2 cells but not in KPad2-SO cells (Fig. 3l).

In aggressive metastatic carcinoma cells, TGFβ induces the expression of *Snai1* (encoding the EMT-transcription factor Snail) coupled with the expression of the fibrogenic factors interleukin-11 (*Il11*), hyaluronan synthase 2 (*Has2*), serpin E1 (*Serpine1*) and others^35,53^. This fibrogenic EMT response to TGFβ depends on RAS–MAPK signaling through RREB1 (Ras response element binding protein 1), and both the EMT and the fibrogenic responses are required for the metastatic outgrowth of aggressive LUAD cells in the lungs^35,36^. The fibrogenic EMT response was present, albeit attenuated in H2087-LCC and M802T4-LCC cells compared to the highly metastatic cell lines A549, derived from a KRAS-mutant human LUAD, and 393T3, derived from an aggressive KP mouse LUAD tumor (Extended Data Fig. 4c,d)^37^. Depletion of RREB1 suppressed these TGFβ gene responses in H2087-LCC cells (Extended Data Fig. 4e,f).

**Extended Data Fig. 5:**
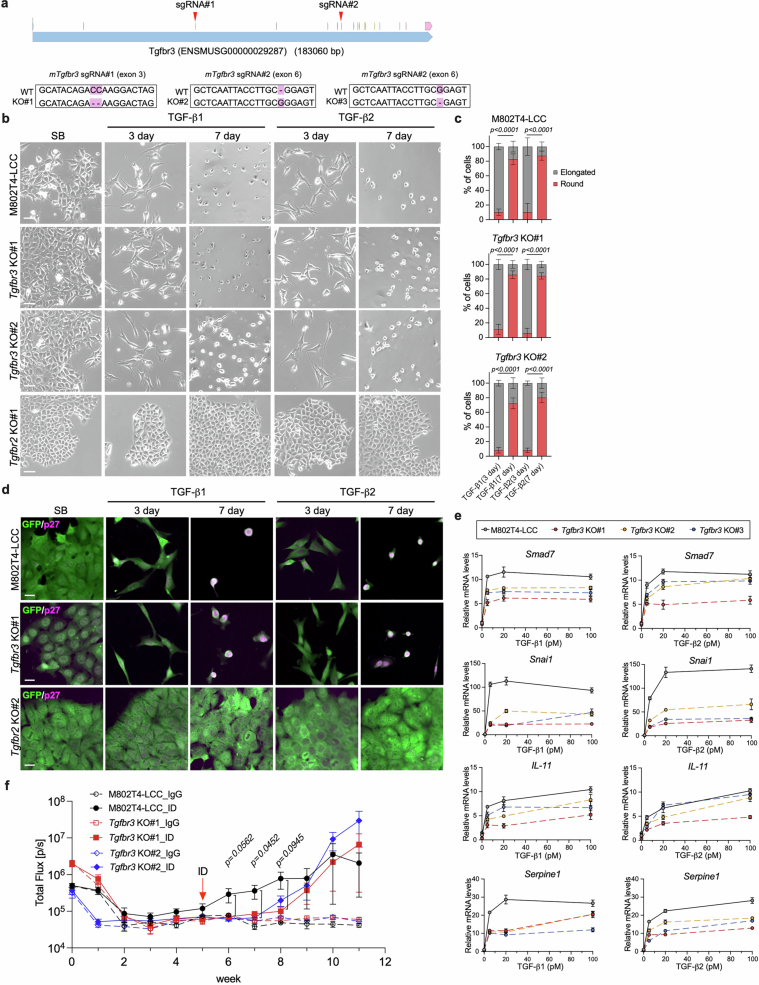
TGF-β1 and TGF-β2 elicit similar EMT responses. **a**, Schematic for sgRNA targeting *Tgfbr3* and sequencing results validating the frame-shifting index within indicated exon for three *Tgfbr3* knockout lines. **b**, Representative bright-field images of wild-type M802T4-LCC cells and indicated KO cells incubated with TGF-β1 or TGF-β2 for 3 days and 7 days. Scale bar: 200 μm. **c**, Quantification of elongated and spheroidal cells upon TGF-β1 or TGF-β2 treatment for 3 days and 7 days. Data are mean ± SD; two-sided unpaired *t*-test. Cells from n = 10 images were analyzed for each condition. **d**, Representative IF images of M802T4-LCC and indicated KO cells incubated with TGF-β1 or TGF-β2 for 3 days and 7 days. GFP^+^ cancer cells (green), p27KIP1 (magenta). Scale bar: 20 μm. **e**, qRT-PCR analysis of indicated gene expression in M802T4-LCC and *Tgfbr3* KO cells treated with different concentrations of TGF-β1 or TGF-β2 for 2 h. Data are mean ± SEM; n = 3 technical replicates. **f**, BLI tracking of wild-type and *Tgfbr3* KO M802T4-LCC cells intravenously inoculated in B6129SF1/J mice followed by antibody-mediated depletion of NK, CD4+ and CD8 + T cells 5 weeks after inoculation. n = 7 mice (IgG groups) or n = 8 mice (ID groups). Data are mean ± SEM. p-values comparing ID groups at indicated time points were shown, one-way ANOVA. Two mice died in week 9 for M802T4-LCC_ID group. Source data

To determine whether these dormant metastasis models react differently to TGFβ1 or TGFβ2, we knocked out *Tgfbr2* (Extended Data Fig. 2k) or *Tgfbr3* (Extended Data Fig. 5a) in M802T4-LCC cells and incubated these cells with TGFβ1 or TGFβ2 for up to 7 days. TGFβ2 induced the same morphologic transitions observed in response to TGFβ1, including a shift from epithelial to spindly morphology followed by a transition to spheroidal morphology (Extended Data Fig. 5b,c) and the expression of p27Kip1 in spheroidal cells (Extended Data Fig. 5d). The knockout of *Tgfbr2* abolished these effects in response to either TGFβ1 or TGFβ2 (Extended Data Fig. 5b–d and refer to Extended Data Fig. 2l). By contrast, *Tgfbr3* knockout did not abolish these effects (Extended Data Fig. 5b–d) and only attenuated to a similar extent the gene responses to TGFβ1 and TGFβ2 (Extended Data Fig. 5e). To examine the effect of knocking out *Tgfbr3* in vivo, we injected wild-type or *Tgfbr3* knockout M802T4-LCC cells into B6129SF1/J mice intravenously and started antibody-mediated depletion of NK cells, CD4^+^ T cells and CD8 + T cells 5 weeks after inoculation. Knockout of *Tgfbr3* resulted in a slight delay in the emergence of metastases (Extended Data Fig. 5f), in sharp contrast with the near complete abolition of metastasis upon the knockout of *Tgfbr2* (Fig. 1m).

In sum, the H2087-LCC, M802T4-LCC and KPad2 LUAD progenitors respond to TGFβ with deployment of an atypical state characterized by a robust EMT transcriptional program, spheroidal morphology lacking actin stress fibers and motility, mild fibrogenic gene responses and strong proliferative quiescence. This contrasts with the TGFβ induction of a persistent EMT associated with fibrogenesis in developmentally more advanced LUAD progenitors, showing that TGFβ triggers distinct EMT responses in primitive (that is, SOX2^+^/NKX2-1^+^) versus late-stage (SOX9^+^) LUAD progenitors.

### TGFβ increases gelsolin expression in LUAD progenitors

**Fig. 4:**
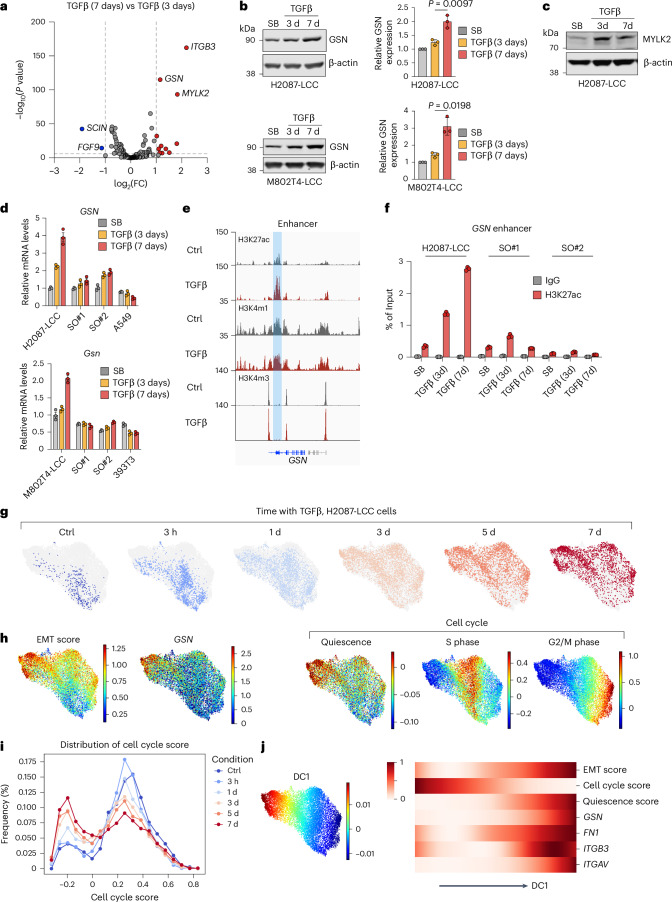
Gelsolin associates with quiescent EMT. **a**, Volcano plot of differentially expressed actin cytoskeleton components and regulators in H2087-LCC cells incubated with TGFβ for 7 days versus 3 days (absolute log_2_(fold change (FC)) > 1). **b**, Western immunoblot analysis (left) and quantification (right) of gelsolin levels in H2087-LCC and M802T4-LCC cells treated with TGFβ for the indicated time periods. β-actin was used as a loading control. Quantification was done with *n* = 3 independent experiments. Data are means; error bars, s.d.; two-sided unpaired *t*-test. **c**, Western immunoblot analysis of MYLK2 levels in H2087-LCC treated with TGFβ for the indicated time periods. β-actin was used as a loading control. **d**, RT–qPCR analysis of *GSN* mRNA levels in the indicated cell lines and TGFβ treatment conditions. Data are means; error bars, s.e.m.; *n* = 3 technical replicates. Results were from one representative experiment of two independent repeats. **e**, Gene track views of H3K27ac, H3K4m1 and H3K4m3 ChIP–seq tags at the *GSN* locus in H2087-LCC cells incubated with TGFβ for 4 days. The shaded region shows histone marks typical of an enhancer. **f**, ChIP–PCR analysis of H3K27ac at the *GSN* enhancer in the indicated cell lines upon incubations with SB or TGFβ for 3 days or 7 days. Data are means; error bars, s.e.m.; *n* = 3 technical replicates. Results were from one representative experiment of two independent repeats. **g**, Force-directed layouts illustrate the differential distribution of cells following the indicated TGFβ treatment, with cells from all conditions (a total of 12,739 cells) shown in the background to facilitate visual inspection. **h**, Single-cell *GSN* expression and EMT, quiescence, S phase and G2/M phase signature scores overlaid on force-directed layouts of the cells from all conditions. **i**, Histogram distributions of EMT, quiescence and cell cycle (including both S phase and G2/M phase) signature scores for cells from each of the indicated TGFβ treatment conditions. **j**, Left panel, force-directed layout displaying the first diffusion component (DC1) values of all cells, represented with a color map; right panel, heatmap showing fitted trends of EMT, cell cycle (including both S phase and G2/M phase), quiescence signature scores, *GSN* and key EMT markers expression along DC1 values. Each feature was standardized across all cells to range between 0 and 1 (rows). Source data

**Extended Data Fig. 6:**
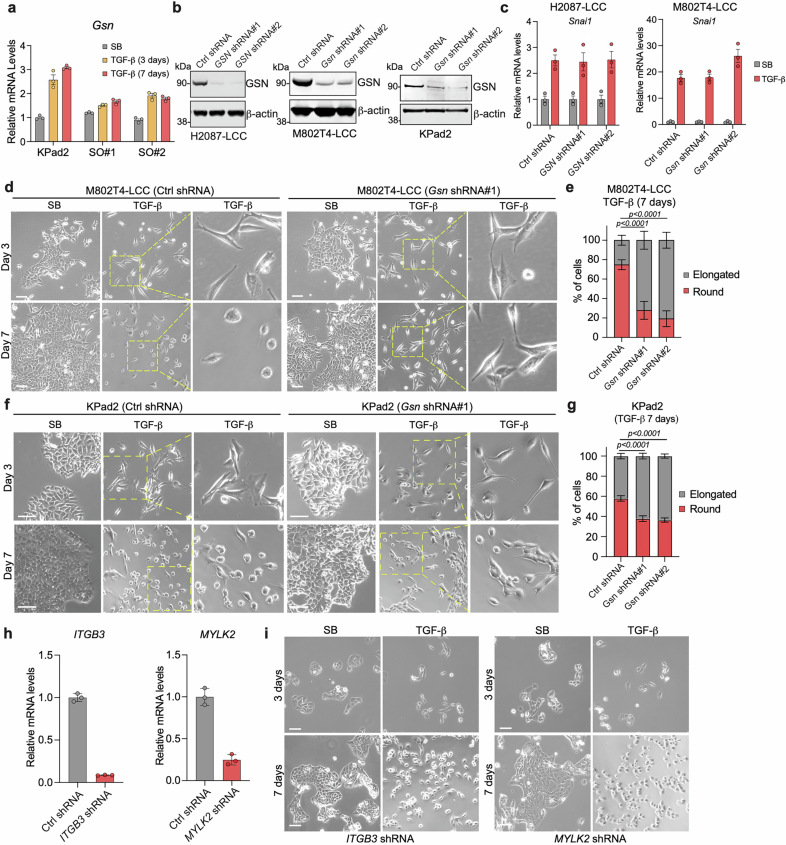
A gelsolin modified EMT. **a**, qRT-PCR analysis of *GSN* mRNA levels in the indicated cell lines and TGF-β treatment conditions. Data are mean ± SEM; n = 3 technical replicates. **b**, Western immunoblot analysis of GSN levels in H2087-LCC, M802T4-LCC and KPad2 cells expressing control shRNA or different shRNAs against *GSN*. β-actin was used as loading control. **c**, qRT-PCR analysis of *Snai1* mRNA levels in H2087-LCC and M802T4-LCC cells expressing the indicated shRNAs incubated with TGF-β for 2 h. Data are mean ± SEM; n = 3 technical replicates. **d**, Representative bright-field images of M802T4-LCC cells expressing control or *GSN* shRNAs and incubated with TGF-β for 3 days or 7 days. Scale bar: 200 μm. **e**, Quantification of elongated and spheroidal cells in the indicated cell lines upon incubation with TGF-β for 7 days. Data are mean ± SD.; results from n = 9 images per condition, two-sided unpaired *t*-test. **f**, Representative bright-field images of KPad2 cells expressing control or *GSN* shRNAs and incubated with TGF-β for 3 days or 7 days. Scale bar: 200 μm. **g**, Quantification of elongated and spheroidal cells in the indicated cell lines upon incubation with TGF-β for 7 days. Data are mean ± SD; results from n = 11 images per condition, two-sided unpaired *t*-test. **h**, qRT-PCR analysis of *ITGB3* and *MYLK2* mRNA levels in H2087-LCC cells expressing the indicated shRNAs. Data are mean ± SEM; n = 3 technical replicates. All qRT-PCR results were from one representative experiment of two independent repeats. **i**, Representative bright field images of H2087-LCC cells expressing the indicated shRNAs and incubated with TGF-β for 3 days or 7 days. Scale bar: 200 μm. Source data

To investigate the basis for the loss of actin stress fibers in LCC cells under persistent exposure to TGFβ, we queried our RNA-seq dataset for differentially expressed actin cytoskeleton components and regulators. *GSN* (gelsolin), *MYLK2* and *ITGB3* emerged as differentially expressed actin cytoskeleton regulatory genes in H2087-LCC cells incubated with TGFβ for 7 days, when most cells had transitioned into the spheroidal morphology, compared with cells incubated with TGFβ for 3 days, when most cells were still spindly (Fig. 4a). In agreement with these results, H2087-LCC and M802T4-LCC cells incubated with TGFβ showed a progressive increase in gelsolin mRNA and protein levels, which was incipient after 3 days and reached twofold to threefold over basal levels after 7 days (Fig. 4b). The protein levels of MYLK2 (Fig. 4c) and integrin β3 (Fig. 2g,h) showed little or no increase at 7 days versus 3 days of incubation with TGFβ. Notably, TGFβ did not increase gelsolin expression in SO derivatives or in the aggressive metastatic LUAD cell lines A549 and 393T3 (Fig. 4d and Extended Data Fig. 6a).

We previously profiled histone 3 (H3) modifications in H2087-LCC cells using chromatin immunoprecipitation with sequencing (ChIP–seq)^18^. H3K4me3, which marks active promoters^54,55^, was enriched near the *GSN* transcription start site independently of TGFβ treatment. H3K4me1 and H3K27ac, which mark active enhancers, were present in a *GSN* intronic region before TGFβ addition. Incubation with TGFβ for 4 days increased the level of H3K27ac in this region (Fig. 4e). ChIP–PCR analysis showed that TGFβ induced an increase in H3K27 acetylation level in this enhancer region in H2087-LCC cells but not in H2087-SO cells derived from spontaneous metastatic outbreaks after incubation with TGFβ for 3 days or 7 days (Fig. 4f).

To further investigate the temporal progression of this atypical EMT, we performed single-cell RNA-seq (scRNA-seq) analysis on H2087-LCC cells treated with TGFβ (Fig. 4g). Throughout the time course of a 7-day TGFβ treatment, cells increasingly transitioned to a mesenchymal state. Cells expressing a high level of gelsolin exhibited the highest EMT score and the highest level of quiescence markers (Fig. 4h,i). Along the top diffusion component (DC1), which corresponds to the highest phenotypic variation, we observed a close association between EMT signature, *GSN* expression and quiescence signature score, and a decline in cell cycle signature score following TGFβ exposure (Fig. 4j). Collectively, these results suggest that TGFβ signaling progressively induces an EMT state associated with gelsolin expression and exit from the cell cycle in LUAD progenitors.

### A gelsolin-modified EMT

**Fig. 5:**
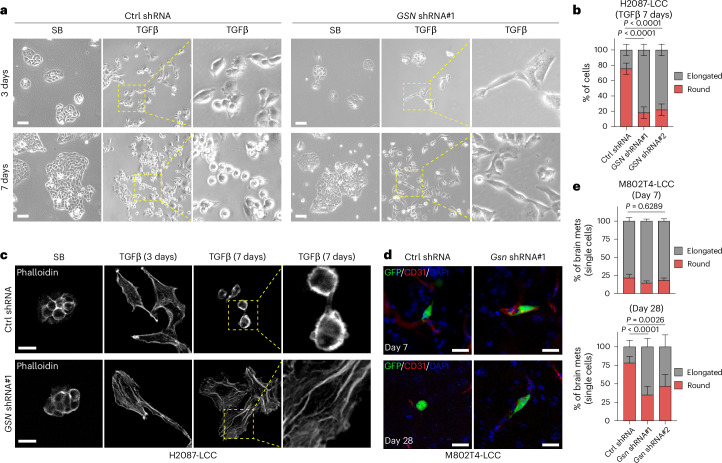
A gelsolin-modified EMT. **a**, Representative bright-field images of H2087-LCC cells expressing control shRNA or *shGSN* and incubated with TGFβ for 3 days or 7 days. Scale bar, 200 μm. **b**, Quantification of elongated versus spheroidal H2087-LCC cells in **a**. Cells from *n* = 12 images (control shRNA) or *n* = 10 images (*GSN* shRNA) were analyzed. Data are means; error bars, s.d.; two-sided unpaired *t*-test. **c**, Representative phalloidin staining of H20878-LCC cells expressing control shRNA or *shGSN* and incubated with TGFβ for 3 days or 7 days. Scale bar, 20 μm. **d**, Representative IF images of M802T4-LCC cells expressing the indicated shRNAs in the brain of Albino-B6 mice 7 days or 28 days after intracardiac inoculation. GFP^+^ cancer cells (green), CD31^+^ capillaries (red), DAPI (blue). Scale bar, 20 μm. **e**, Quantification of elongated versus spheroidal M802T4-LCC cells in the brain parenchyma of Albino-B6 mice that were intracardially inoculated with these cells 7 days or 28 days prior. Results from *n* = 4 mice (day 7) or *n* = 5 mice (day 28) per group. Chi-squared test. Data are means; error bars, s.e.m. Source data

Gelsolin promotes actin cytoskeleton turnover by severing and capping of actin filaments^56,57^. The observed loss of actin stress fibers under TGFβ stimulation suggested a role of gelsolin in this transition. To investigate this possibility, we knocked down gelsolin expression in H2087-LCC, M802T4-LCC and KPad2 cells (Extended Data Fig. 6b), which did not inhibit the induction of *Snai1* by TGFβ (Extended Data Fig. 6c). The depletion of gelsolin did not interfere with the initial induction of a spindly cell morphology by TGFβ, but it consistently prevented the transition to spheroidal morphology after prolonged incubation with TGFβ (Fig. 5a,b and Extended Data Fig. 6d–g). Phalloidin staining of actin filaments confirmed that gelsolin knockdown inhibited the TGFβ-induced transition of actin stress fibers to cortical actin filaments (Fig. 5c). Unlike the gelsolin knockdown, knockdown of *ITGB3* or *MYLK2* did not affect the transition of H2087-LCC cell morphology from spindly to spheroidal during incubation with TGFβ (Extended Data Fig. 6h,i).

To determine the role of gelsolin in the transition of disseminated LUAD cells to a spheroidal morphology in vivo, we performed quantitative morphometry in M802T4-LCC cells disseminated to the brain. The knockdown of gelsolin had no effect on the predominantly elongated morphology of recently extravasated cells compared to controls 1 week after intracardiac inoculation (Fig. 5d,e). However, 4 weeks after inoculation, the proportion of spheroidal M802T4-LCC cells remaining in the brain was significantly reduced in gelsolin knockdown cells compared to control cells (Fig. 5d,e). Collectively, these results identify gelsolin as a critical mediator of the morphological transition of dormancy-competent LUAD cells from an elongated, stress-fiber-rich mesenchymal state during the early phase of a TGFβ response to a cortical actin-rich mesenchymal state after a prolonged exposure to TGFβ.

### An immune-evasive decrease in cell stiffness

The cytoskeleton determines the stiffness of a cell^58^, and a high level of stiffness in cancer cells favors the formation of the cytotoxic synapse and consequent killing by CTLs and NK cells^29,31–33^. The dramatic switch from a stress-fiber-rich state to a spheroidal, cortical actin-rich state induced by TGFβ in dormant metastatic cells raised the possibility that this transition regulates the susceptibility of the cells to immune mechanosurveillance during long-term dormancy.

**Fig. 6:**
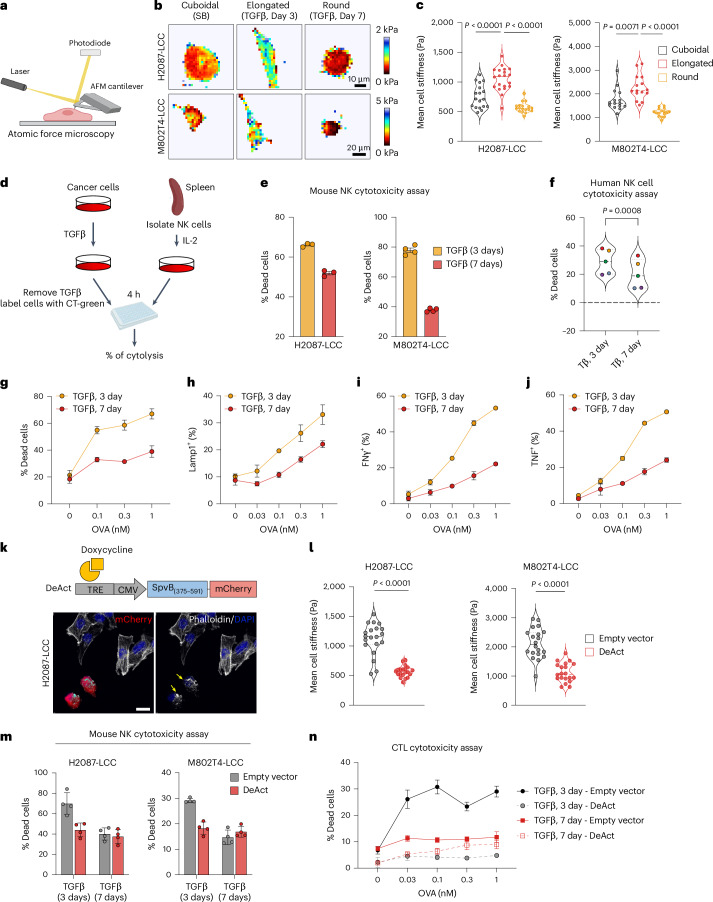
An immune-evasive decrease in cell stiffness. **a**, Schematic representation of AFM determination of the biomechanical stiffness of cells (created with Biorender.com). **b**, Force maps of representative cells, with Young’s modulus value (kPa) indicated in pseudocolor. **c**, Mean cell stiffness of elongated and spheroidal H2087-LCC or M802T4-LCC cells in response to TGFβ treatment for 3 days and 7 days. Violin distributions are centered around the median (dashed line), with quartile ranges displayed above and below (dotted lines). *n* = 18 cells per condition for H2087-LCC cells; *n* = 15 cells per condition for M802T4-LCC cells. Two-sided unpaired *t*-test. **d**, Schematic of in vitro mouse NK cell cytotoxicity assay (created with Biorender.com). **e**, H2087-LCC and M802T4-LCC cells were incubated with TGFβ for the indicated time periods and admixed with IL-2-activated mouse NK cells. Data are means; error bars, s.e.m.; *n* = 3 technical replicates (H2087-LCC) or *n* = 4 technical replicates (M802T4-LCC). **f**, H2087-LCC cells were incubated with TGFβ for the indicated time periods and admixed with NK cells derived from human peripheral blood from *n* = 5 different donors. After 4 h of coincubation, the percentage of dead cancer cells was determined by flow cytometry. Each dot is the mean of four technical replicates; colors indicate samples derived from the same donor. Two-sided paired *t*-test. **g**, M802T4-LCC cells incubated with TGFβ for 3 days or 7 days were loaded with increasing concentrations of OVA peptide and then mixed with OT1 CTLs. Cancer cell lysis was quantified after 5 h. Data are means; error bars, s.d.; *n* = 3 technical replicates. **h**, CTL degranulation as determined by surface exposure of Lamp1 5 h after mixing OT1 CTLs with OVA-loaded M802T4-LCC cells. Data are means; error bars, s.d.; *n* = 3 technical replicates. **i**,**j**, Production of IFNγ and TNF, measured by intracellular immunostaining of CTLs 5 h after mixing with the indicated M802T4-LCC cells. Data are means; error bars, s.d.; *n* = 3 technical replicates. **k**, Schematic representation of a vector driving Dox-inducible co-expression of the DeAct SpvB_375–591_ and an mCherry marker. Representative images of H2087-LCC cells incubated with doxycycline for 24 h to induce DeAct and mCherry expression in transduced H2087-LCC cells admixed with non-transduced cells. Phalloidin staining demonstrates the absence of actin stress fibers in DeAct-expressing cells. **l**, Mean cell stiffness in H2087-LCC and M802T4 cells incubated with TGFβ for 3 days to induce EMT and express control vector or DeAct for 24 h before analysis. *n* = 20 cells per condition; two-sided unpaired *t*-test. **m**, cytotoxic effect of mouse NK cells on the indicated cancer cells incubated with TGFβ for 3 days or 7 days and expressing control vector or DeAct 24 h before analysis. Data are means; error bars, s.e.m.; *n* = 4 technical replicates. **n**, cytotoxic effect of CTLs on M802T4-LCC cells incubated with TGFβ for 3 days or 7 days and expressing control vector or DeAct 24 h before analysis. Data are means; error bars, s.d.; *n* = 3 technical replicates. All in vitro cytotoxic assay results were from one representative experiment of two independent repeats. Source data

**Extended Data Fig. 7:**
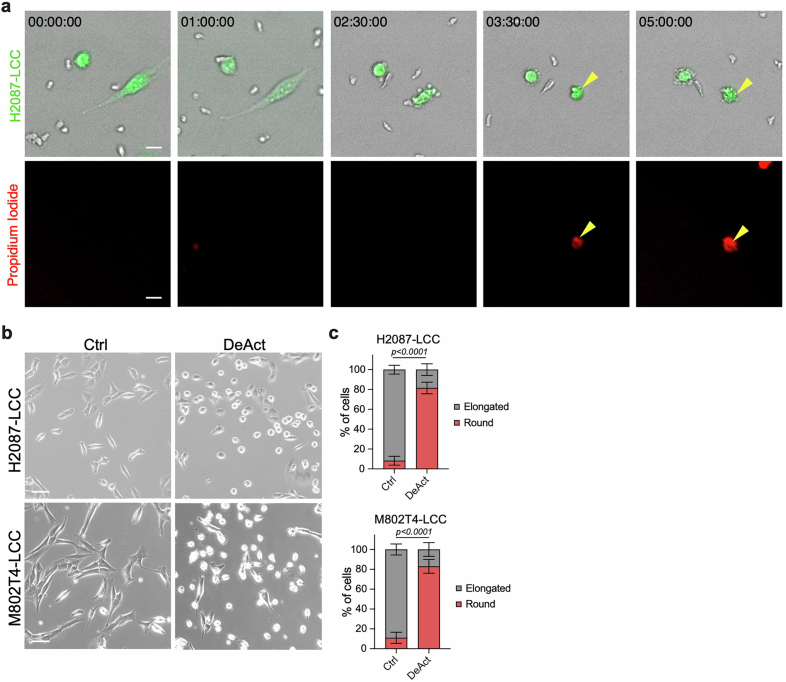
An immune evasive decrease in cell stiffness. **a**, H2087-LCC cells were incubated with TGF-β for 5 days to generate a mixed of cells with elongated or spheroidal morphology. The cells were then admixed with IL-2-activated mouse NK cells in the presence of propidium iodide, which stains the nuclear DNA of dead cells. Shown are still frames from time-lapse imaging of H2087-LCC and NK cell cocultures. Arrowhead, an elongated cancer cell undergoing death near a spheroidal cancer cells that survives NK attack. Scale bar: 50 μm. **b**, Representative bright-field images of H2087-LCC and M802T4 cells incubated with TGF-β for 3 days to induce an EMT and express control vector or DeAct for 24 h. **c**, Quantification of elongated and spheroidal cells for each condition in **b**. Data are mean ± SD.; results from n = 10 images per condition, two-sided unpaired *t*-test. Source data

We used atomic force microscopy (AFM) to determine the surface tension of LUAD cells under different TGFβ treatment conditions in culture (Fig. 6a). LCC cells gained stiffness as they adopted a spindly morphology upon incubation with TGFβ for 3 days. The level of cell stiffness then declined as the cells adopted a spheroidal morphology under continued stimulation by TGFβ (Fig. 6b,c). To determine whether this transition to a soft morphology was associated with a change in cell susceptibility to immune-mediated killing, we treated H2087-LCC and M802T4-LCC cells with TGFβ and co-cultured the cells with mouse NK cells (Fig. 6d). Cells incubated with TGFβ for 3 days were more susceptible to NK-cell-mediated killing than cells incubated for 7 days (Fig. 6e). Similar results were obtained when H2087-LCC cells were co-cultured with human NK cells isolated from peripheral blood from different donors (Fig. 6f). Live imaging of H2087-LCC cell cultures incubated with TGFβ for 5 days, which included a mix of spindly and spheroidal cells, revealed that NK cells interacted with cells in both morphologies but were more effective at killing the spindly cancer cells (Extended Data Fig. 7a and Supplementary Video 2).

To determine whether the TGFβ-dependent change in cell morphology was associated with a difference in killing by CTLs, we incubated M802T4-LCC cells with TGFβ for 3 days or 7 days before adding ovalbumin_257–264_ peptide (OVA), which binds to the MHC-I molecule H-2K^b^ and renders cells susceptible to killing by CTLs expressing the OVA T cell receptor OT1 (ref. ^59^). When co-cultured with OT1 CTLs, cancer cells were more susceptible to killing after incubation with TGFβ for 3 days than for 7 days (Fig. 6g). Moreover, cells incubated with TGFβ for 3 days induced stronger CTL activation, as indicated by increased degranulation (Fig. 6h) and increased production of the inflammatory cytokines interferon-γ (IFNγ) and tumor necrosis factor (TNF) (Fig. 6i,j).

The ADP-ribosyl transferase domain (referred to as DeAct) of *Salmonella enterica* SpvB drives the disassembly of filamentous actin through ADP-ribosylation of actin on Arg177 (ref. ^60^). To establish causality between actin cytoskeleton depolymerization and evasion of immune surveillance, we engineered H2087-LCC and M802T4-LCC cells with inducible DeAct expression as a genetically encoded actin disassembly tool (Fig. 6k). During the early phase (day 3) of incubation with TGFβ, cells expressing DeAct (marked by mCherry) for 24 h showed a lack of actin stress fibers and significant change of morphology relative to controls (Fig. 6k and Extended Data Fig. 7b,c). DeAct-expressing cells exhibited a dramatic reduction in cell stiffness (Fig. 6l) and were less sensitive to both NK-cell-mediated and CTL-mediated killing (Fig. 6m,n). These results indicate that actin cytoskeleton-dependent cell stiffness regulates immune surveillance in these cells.

### Gelsolin protects dormant metastasis from mechanosurveillance

**Fig. 7:**
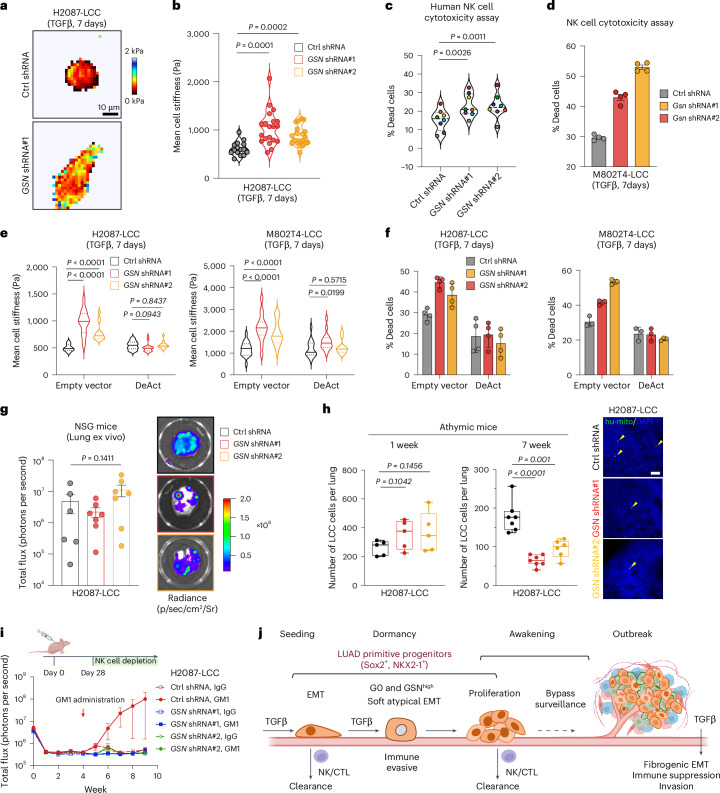
Gelsolin protects LUAD progenitors from mechanosurveillance. **a**, Force maps of representative H2087-LCC cells harboring control or *GSN-*targeting shRNAs and incubated with TGFβ for 7 days. **b**, Mean cell stiffness of H2087-LCC cells harboring the indicated shRNAs and incubated with TGFβ treatment for 7 days. Violin distributions are centered around the median (dashed line), with quartile ranges displayed above and below (dotted lines). *n* = 15 cells (control shRNA) or *n* = 20 cells (*GSN* shRNA); two-sided unpaired *t*-test. **c**, Cytotoxic effect of human NK cells on H2087-LCC cells harboring the indicated shRNAs and incubated with TGFβ for 7 days. Each dot is the mean of four technical replicates. Colors indicate samples derived from the same donor (*n* = 8 donors). Two-sided paired *t*-test. **d**, Cytotoxic effect of mouse NK cytotoxicity on M802T4-LCC cells expressing the indicated shRNAs and incubated with TGFβ for 7 days. Data are means; error bars, s.e.m.; *n* = 4 technical replicates. Results were from one representative experiment of three independent repeats. **e**, Mean cell stiffness of H2087-LCC and M802T4-LCC cells harboring the indicated shRNA and expressing control vector or DeAct. The cells were incubated with TGFβ treatment for 7 days. *n* = 15 cells per condition, two-sided unpaired *t*-test. **f**, Cytotoxic effect of mouse NK cells on H2087-LCC and M802T4-LCC cells harboring the indicated shRNA and expressing control vector or DeAct. The cells were incubated with TGFβ for 7 days. Data are means; error bars, s.e.m.; *n* = 4 (H2087-LCC) or *n* = 3 (M802T4-LCC) technical replicates. **g**, Ex vivo BLI signal and representative images of lungs from NSG mice that were intravenously injected with H2087-LCC cells expressing the indicated shRNAs. Data are means; error bars, s.e.m.; *n* = 6 (control shRNA) or *n* = 7 (*GSN* shRNA) mice per group; one-way ANOVA (*P* = 0.1411). **h**, Quantification of residual H2087-LCC cells in the lungs of athymic mice 1 week and 7 weeks after intravenous inoculation. *n* = 5 (1 week) or *n* = 7 mice (7 weeks) per group; two-sided unpaired *t*-test. Box plots show median ± interquartile range; whiskers indicate minima and maxima. Representative IF images of residual H2087-LCC cells 7 weeks after inoculation are shown on the right. Arrowheads indicated disseminated cancer cells. Scale bar, 100 μm. **i**, H2087-LCC cells expressing shRNA against *GSN* were intravenously inoculated into athymic mice. The mice were administered anti-asialo-GM1 antibody to deplete NK cells and allow the metastatic outgrowth of remaining disseminated cancer cells. IgG control or GM1 antibody administration started 4 weeks after cancer cell inoculation. Tumor burden was tracked by BLI analysis. Data are means; error bars, s.e.m.; *n* = 6 (IgG) or *n* = 7 (GM1) mice per group. **j**. Schematic summary of how TGFβ protects LUAD primitive progenitors (SOX2^+^ or NKX2-1^+^) cells from immune mechanosurveillance through gelsolin-modified EMT program (created with Biorender.com). Source data

**Extended Data Fig. 8:**
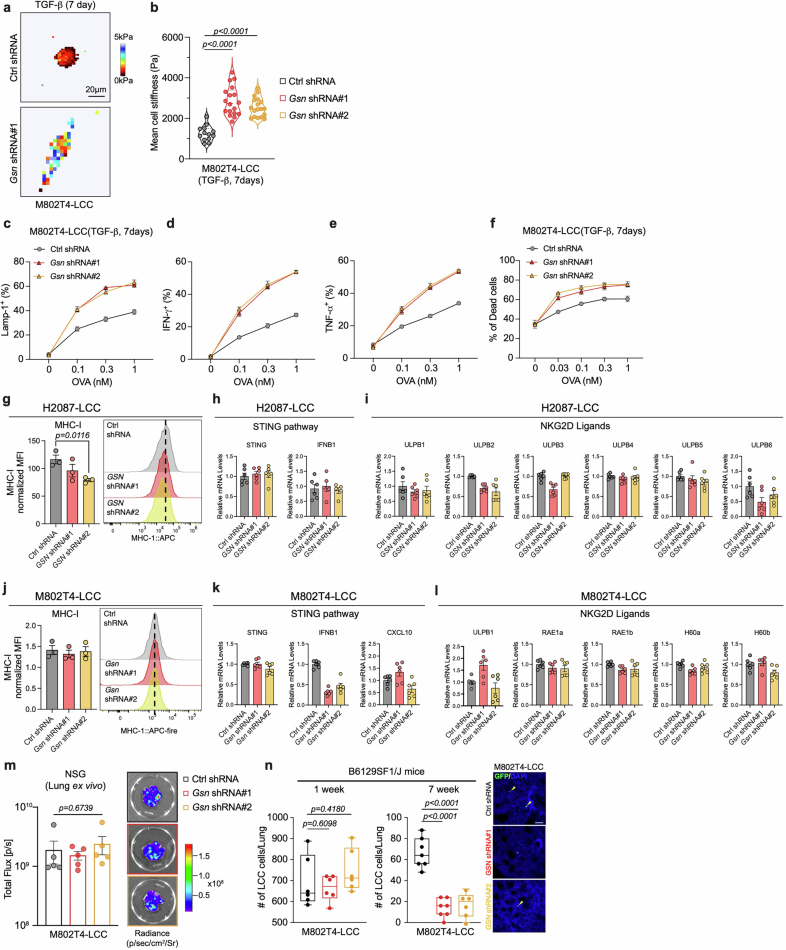
Gelsolin protects LUAD progenitors from mechanosurveillance. **a**, Force maps of representative M802T4-LCC cells expressing the indicated shRNAs and incubated with TGF-β for 7 days. **b**, Mean cell stiffness of M802T4-LCC cells expressing the indicated shRNA and incubated with TGF-β for 7 days. n = 13 (control shRNA) or 18 (*Gsn* shRNAs) cells per condition; two-sided unpaired *t*-test. **c**, M802T4-LCC cells expressing the indicated shRNA were incubated with TGF-β for 7 days and loaded with OVA peptide. The cancer cells were then co-incubated with OT1 CTLs for 5 h. CTL degranulation was determined by surface exposure of Lamp1. **d** and **e**, Production of IFN-γ and TNF-α, measured by intracellular immunostaining of CTLs from the experiments in D. **f**, cytotoxic effect of OT1 CTLs on M802T4-LCC cells expressing the indicated shRNA and incubated with TGF-β for 7 days. Data are mean ± SD; n = 3 technical replicates. Results from one representative experiment of two independent repeats were shown. **g**, Flow cytometry analysis of MHC-I expression in H2087-LCC cells expressing indicated shRNA. Data are mean ± SEM, n = 3 biological replicates, RM One-way-ANOVA with Dunnett post-test. **h** and **i**, qRT-PCR analysis of STING pathway genes and NKG2D ligands in H2087-LCC cells expressing indicated shRNA. Data are mean ± SEM, results are from two independent experiments combined with 3 technical replicates each. One-way-ANOVA with Dunnett post-test. **j**, Flow cytometry analysis of MHC-I expression in M802T4-LCC cells expressing indicated shRNA. Data are mean ± SEM, n = 3 biological replicates, RM One-way-ANOVA with Dunnett post-test. **k** and **l**, qRT-PCR analysis of STING pathway genes and NKG2D ligands in M802T4-LCC cells expressing indicated shRNA. Data are mean ± SEM, results are from two independent experiments combined with 3 technical replicates each. One-way-ANOVA with Dunnett post-test. **m**, Ex vivo BLI signal and representative images lungs from NSG mice that were intravenously injected with M802T4-LCC cell expressing the indicated shRNA. Data are mean ± SEM; n = 5 mice per group; one-way ANOVA. **n**, Quantification of residual M802T4-LCC cells in the lungs 1 week and 7 weeks after intravenous inoculation into B6129SF1/J mice. n = 6 (1 week) or 7 mice (7 week) per group; two-sided unpaired *t*-test. Box plots show median ± interquartile range; whiskers indicate minima and maxima. Representative IF images of residual M802T4-LCC cells 7 weeks after inoculation shown on the right. Scale bar: 100 μm. Source data

Next, we tested the hypothesis that TGFβ allows dormant progenitor cells to evade mechanosurveillance through a gelsolin-mediated morphological transition. AFM measurements showed that knockdown of gelsolin caused a persistence of stiffness in cells incubated with TGFβ for 7 days, both in the H2087-LCC (Fig. 7a,b) and M802T4-LCC models (Extended Data Fig. 8a,b), and rendered these cells more sensitive to NK-cell-mediated killing (Fig. 7c,d). Similarly, knocking down gelsolin in M802T4-LCC cells drove stronger degranulation of CTLs and cytokine production (Extended Data Fig. 8c–e) and resulted in increased killing of cancer cells (Extended Data Fig. 8f). We did not detect significant and consistent changes in the cell surface level of MHC-I molecules, the expression of STING pathway components or NK cell receptor ligands in TGFβ-treated control versus *GSN* knockdown cells that could account for their increased susceptibility to cytotoxic lymphocytes (Extended Data Fig. 8g–l). Importantly, expression of DeAct reversed the increased susceptibility to NK-cell-mediated killing conferred by gelsolin knockdown in both H2087-LCC and M802T4-LCC cells (Fig. 7e,f).

To explore the role of gelsolin in mediating immune evasion of dormant metastatic cells in vivo, we intravenously inoculated H2087-LCC cells bearing control shRNA or shRNA against *GSN* into NSG mice. We observed no effect of gelsolin knockdown on lung colonization, indicating that gelsolin is not critical for metastatic outgrowth in the absence of immune surveillance (Fig. 7g). We then inoculated these cells into athymic mice and determined the number of disseminated cancer cells in the lungs. Gelsolin knockdown did not significantly alter the number of cancer cells seeded to the lungs 1 week after injection but decreased the number of cancer cells surviving in the lungs 7 weeks post injection (Fig. 7h). Depleting NK cells in athymic mice 4 weeks after inoculation allows the outgrowth of metastatic colonies by disseminated H2087-LCC cells, indicating that LCC cells withstand NK-cell-mediated killing in the dormant state and can subsequently grow after this immune pressure is relieved. By contrast, no metastases emerged after NK depletion in mice harboring gelsolin-depleted H2087-LCC cells (Fig. 7i). These results suggest that gelsolin is required for resistance to immune surveillance by NK cells during the dormant phase. Similar results were observed with the M802T4-LCC model (Extended Data Fig. 8m,n).

## Discussion

During the active colonization of distant sites, cancer cells are thought to adopt a stiff migratory EMT phenotype that enables their extravasation and invasion for metastatic dormancy and eventual outbreak. However, this stiff phenotype can sensitize metastatic cells to killing by CTLs and NK cells^33^. Focusing on the immediate stages after dissemination, our results show that LUAD primitive progenitors entering dormancy in response to TGFβ initially display the morphological, transcriptional, cytoskeletal and migratory traits of an archetypical EMT but subsequently transition into a soft, poorly migratory phenotype lacking actin stress fibers while retaining a strong EMT transcriptional program. This transition is mediated by a progressive increase in gelsolin expression, which remodels the actin cytoskeleton. The resulting loss of stiffness allows these cells to better evade immune surveillance and survive as metastatic progenitors during prolonged dormancy (Fig. 7j).

Our results add further evidence that entry into dormancy is not a passive response of metastatic cells to growth-inhibitory cues in host organs but rather is an active process. Malignant primitive progenitors appear to navigate this crucial step in disease progression by limiting their vulnerability to immune surveillance, in part by diminishing their mechanical susceptibility. Softer cancer cells are more resistant to immune-mediated clearance both in vitro and in vivo^33,61,62^. The rapidity and prevalence of cell rounding during TGFβ-induced dormancy highlight the relevance of mechanosurveillance and of its evasion for the survival of dormant disseminated LUAD cells. It will be interesting to determine whether gelsolin-mediated cell softening or a functionally analogous mechanism supports cancer cell survival during dormancy in other types of cancer. Of note, the gelsolin family includes additional members (villin, advillin, capG) that have been linked to tumorigenesis and EMT^63–65^.

EMT represents a spectrum of phenotypic states, and carcinomas harbor cancer cell populations that display a range of EMT states associated with different levels of metastatic aggressiveness^66^. In contrast to the partial EMTs described in previous studies, the dormancy-associated EMT of LUAD primitive progenitors is characterized by high expression of an extensive ensemble of mesenchymal markers and downregulation of epithelial markers, thus resembling a full EMT except for the gelsolin-mediated removal of actin stress fibers, resulting in a loss of spindly cell morphology. The dormancy-associated EMT also appears to be distinct from the amoeboid state described in highly proliferative melanoma, hepatocellular carcinoma and breast carcinoma cells^67^, which is usually driven by myosin light chain 2 phosphorylation^10^. The EMT response induced by TGFβ in SOX2^+^ and NKX2-1^+^ LUAD progenitors also contrasts with the EMT induced by TGFβ in the metastatically aggressive progeny of these cells, where SOX9^+^ late-stage progenitors are predominant. These progenies respond to TGFβ with an archetypical EMT that is coupled to expression of fibrogenic factors supporting metastatic outgrowth and invasion^35,36^ (Fig. 7j). During each stage of the metastatic process, disseminated cancer cells must undergo dramatic biomechanical adaptations to survive under physical stress. The present and previous findings suggest that as disseminated metastasis-initiating cells progress into overt metastases and their progeny deploy different stages in a developmental continuum^17^, each stage in this continuum presents a distinct EMT program to fulfill different pro-metastatic functions.

Our findings expand the concept that dormancy proactively protects disseminated cancer cells. LUAD metastatic progenitors entering dormancy downregulate not only MHC molecules^21–24^, NK cell ligands^15^ and STING signaling^18^ but also cell stiffness as a set of immune-evasive mechanisms. Gelsolin-mediated evasion of immune surveillance unexpectedly reveals a distinct strategy for metastatic progenitors to alleviate a liability associated with a full mesenchymal morphology, protecting these cells during the highly vulnerable period of solitary persistence^3,26^. These observations illustrate the complex dynamics of TGFβ in molding EMT responses to ultimately promote metastasis. Perturbations that diminish this protection led to depletion of dormant disseminated disease in our models, suggesting a basis for new approaches to treat cryptic metastasis.

## Methods

### Animal studies

All animal experiments were performed in accordance with protocols approved by the Memorial Sloan Kettering Cancer Center (MSKCC) Institutional Animal Care and Use Committee (protocol 99-09-032). Maximum tumor burden was not exceeded in the animal experiments. Athymic nude mice were obtained from Envigo (strain no. 069) or Charles River Laboratories (strain no. 490). NSG (NOD.Cg-*Prkdc*^*scid*^*IL2rg*^*tm1Wjl*^/SzJ; strain no. 005557), B6129SF1/J (strain no. 101043) and B6(Cg)-*Tyr*^*c-2J*^/J (B6-albino; strain no. 000058) mouse strains were obtained from the Jackson Laboratory. Female mice 6–8 weeks of age were used for in vivo studies; 2–6-month-old male and female OT1 αβTCR transgenic mice (Jackson Laboratories; strain no. 003831) were used to generate OT1 CTLs for in vitro assays.

For brain metastasis assays, 1 × 10^5^ cells were resuspended in 100 µl of PBS and intracardially injected into the right ventricle. Lung colonization assays were performed by injecting 1 × 10^5^ or 5 × 10^4^ cells into the lateral tail vein of mice. Metastatic burden was monitored by bioluminescence imaging (BLI) using retro-orbital injection of D-luciferin (150 mg kg^−1^) and an IVIS Spectrum Xenogen instrument (PerkinElmer). Data were analyzed using Living Image software (v.4.5; PerkinElmer).

For experiments with cells engineered with a TGFβ reporter, mice were maintained on a regular diet and switched to a diet of doxycycline food pellets (2,500 mg kg^−1^; Envigo) 2 weeks before dissecting organs for analysis.

For NK cell depletion experiments in athymic nude mice, 33 μg of anti-asialo-GM1 antibody (Wako Chemical, 986-10001) was injected intraperitoneally per mouse once every 5 days. For NK, CD4^+^ or CD8^+^ T cell depletion assays in B6129SF1/J and B6(Cg)-*Tyr*^*c-2J*^/J mice, 200 μg *InVivo*Mab anti-mouse NK1.1 antibody (clone PK136; BioXCell, BE0036), CD4 antibody (clone GK1.5; BioXCell, BE0003-1), CD8α antibody (clone 53-6.7; BioXCell, BE0004-1) or IgG2a control (clone 2A3; BioXCell, BE0089) was injected intraperitoneally in each mouse once weekly. Mice were randomly assigned to control or treatment groups.

### Cell culture

H2087-LCC cells and derivatives from SO of H2087-LCC cells were cultured in media as previously described^18^. Mouse lung cancer cell line 802T4 was a gift from T. Jacks (Koch Institute, MIT)^37^. M802T4-LCC, KPad2, SO derivatives, 393T3 and A549 (ref. ^68^) cells were cultured in RPMI 1640 media supplemented with 10% FBS, 2 mM glutamine, 100 IU ml^−1^ penicillin–streptomycin and 1 µg ml^−1^ amphotericin B. The 293T cells were cultured in DMEM supplemented with 10% FBS and 2 mM glutamine. All cell lines tested negative for mycoplasma contamination.

For isolation of M802T4-LCC cells, 802T4 cells (1 × 10^5^) expressing a lentiviral vector encoding firefly luciferase and GFP in combination with a puromycin antibiotic resistance marker were intravenously injected into B6129SF1/J mice. Cancer cell colony growth was tracked by BLI. Lungs from BLI-negative mice were resected under sterile conditions and dissociated using the Lung Dissociation Kit (Miltenyl Biotec, 130-095-927) following the manufacturer’s instructions. Cells were then resuspended in culture conditions and allowed to attach in a 15-cm dish. Cancer cells from these BLI-negative lungs were selected with 2.5 μg ml^−1^ of puromycin (Gibco). The KPad2 cell line and its SO derivatives were generated as previously described^18^.

For analysis of TGFβ responses, cells were cultured in media containing 2% FBS and incubated with SB-505124 (2.5 μM; Millipore Sigma, S4696-5MG), 100 pM TGFβ1 (R&D Systems) or 100 pM TGFβ2 (R&D Systems, 302-B2-002) for the indicated period of time.

For analysis of Wnt-3a response, cells were cultured in media containing 2% FBS and treated with recombinant human Wnt-3a protein (R&D Systems, 5036-WN-010) at a final concentration of 200 ng ml^−1^ for 2 h.

### Gene knockout, knockdown and overexpression constructs

CRISPR-mediated knockouts were generated by cloning sgRNAs into the Guide-it CRISPR–Cas9 vector (Red, TaKaRa, 632602), transfecting the construct into cells and isolating and expanding the cells with knockouts from single-cell colonies. Sequences of sgRNA oligonucleotides are listed in Supplementary Table 3.

Stable knockdowns of *GSN* in H2087-LCC cells were generated using two independent short hairpin RNAs (shRNAs) purchased from Millipore Sigma (MISSION Predesigned shRNA clone, shRNA1: TRC Clone ID TRCN0000029725; shRNA2: TRC Clone ID TRCN0000029728). Non-mammalian shRNA was used as a control (Millipore Sigma, SHC002). Stable knockdowns of *Gsn* in M802T4-LCC cells were generated using two independent shRNAs purchased from Horizon Discovery (SMARTvector Lentiviral shRNA, shRNA1: Clone ID V3SVMM08_10907046; shRNA2: Clone ID V3SVMM08_11757555). Non-targeting shRNA was used as a control (Horizon Discovery, VSC11715)

For DeAct-SpVB expression, the DHRFdd-SpvB (*Salmonella* SpvB_375–591_) sequence was amplified by PCR from pTetON-DHFRdd-SpvB; CMV-mCherry (Addgene, plasmid no. 89463). PCR-amplified products were ligated to XmalI/MluI-digested pLVX-TRE3G-mCherry (Takara, 631360), a tetracycline-inducible lentiviral expression vector. For all experiments, expression of DeAct was induced 24 h before analysis.

### mScarlet-p27K^−^ reporter

The pCDH-EF1-mVenus-p27K^−^ plasmid (Addgene, 176651) was altered to express mScarlet instead of mVenus to facilitate detection in our GFP^+^ models. The plasmid was digested with XbaI and XmaI enzymes (New England Biolabs) to remove the mVenus cassette and gel-purified. The mScarlet fragment was synthesized as a gblock (Integrated DNA Technologies), and blunt overhangs were added by PCR to match the backbone vector. After gel purification, the new mScarlet fragment was ligated into the open backbone via Gibson Assembly (NEB).

### Immunofluorescence and live imaging

For immunofluorescence staining of tissue samples, organs were fixed in 4% paraformaldehyde overnight at 4 °C and then washed twice with PBS. Organs were cryo-protected by immersion in 30% sucrose, then mounted using OCT (Sakura, 4583) on a sliding microtome with a platform freezing unit (Thermo Fisher Scientific, Microm KS-34 and Microm HM-450). Then, 80 μm sections were cut and stored in anti-freezing solution (30% v/v ethylene glycol, 30% glycerol v/v in PBS) at −20 °C. Floating sections representative of the entire organ were permeabilized by washing in 0.25% Triton X-100 in PBS with (PBS-Tr) three times, followed by incubation for 1 h in blocking buffer containing 10% normal goat serum (Life Technologies, 50062Z), 2% BSA (Fisher Scientific, BP9706100) and 0.25% Triton X-100. Sections were then incubated with primary antibodies diluted in blocking buffer overnight at 4 °C. After washing with PBS-Tr six times, sections were incubated in fluorophore-conjugated secondary antibodies for 2 h. Sections were washed with PBS-Tr and then PBS three times each, followed by staining with 4’,6-diamidino-2-phenylindole (DAPI) nuclear dye (Thermo Fisher Scientific, D3571) for 5 min and three additional washes with PBS. Sections were transferred onto slides and mounted using ProLong diamond antifade mountant (Life Technology, P36970).

For immunofluorescence staining of cells in culture, cells plated in Nunc Lab-Tek II chamber slides (Thermo Fisher, 154453) were fixed for 15 min at room temperature (20–25 °C) in 4% paraformaldehyde and then washed three times with PBS. Samples were incubated for 1 h in blocking buffer containing 10% normal goat serum (Life Technologies, 50062Z), 2% BSA (Fisher Scientific, BP9706100) and 0.25% Triton X-100. Cells were then incubated in primary antibodies, diluted in blocking buffer, overnight at 4 °C or for 2 h at room temperature. After washing with PBS three times, cells were incubated in fluorophore-conjugated secondary antibodies for 1 h at room temperature. Cells were washed with PBS three times, followed by staining with DAPI (Thermo Fisher Scientific, D3571) for 5 min. The EdU incorporation assay was performed with Click-iT Plus EdU Cell Proliferation Kit for Imaging, Alexa Fluor 594 dye (Thermo Fisher Scientific, C10639). Cells were incubated with 10 μM EdU for 1 h, following the manufacturer’s instructions.

Immunofluorescent images were acquired with a Zeiss Axio Imager Z1 microscope or an SP5 confocal microscope (Leica Microsystems). Fluorescent intensity was analyzed with ImageJ software. Antibodies used for immunofluorescence are listed in Supplementary Table 4.

For time-lapse live imaging, 2 × 10^4^ cancer cells were plated in six-well plates and incubated with TGFβ for 5 days. Plates were imaged at 6 min intervals using a Zeiss Zen epifluorescence microscope at ×10 magnification for 48 h. For live imaging of NK-cell-mediated killing, cancer cells were incubated with TGFβ for 5 days and labeled with CellTracker Green CMFDA Dye (Thermo Fisher Scientific, C7025). After washing, cells were mixed with mouse NK cells at a 1:4 carcinoma-to-NK cell ratio in the presence of 1.5 µM propidium iodide. Plates were imaged at 6 min intervals using a Zeiss Zen epifluorescence microscope at ×10 magnification for 5 h.

### RNA sequencing and data analysis

Total RNA purified from cells was quantified by Ribogreen and quality assessed by Agilent BioAnalyzer. A total of 500 ng of RNA with an RNA integrity number of >9.5 from each sample was used for library construction with TruSeq RNA Sample Prep Kit v2 (Illumina) according to the manufacturer’s instructions. Multiplexed sequencing libraries were run on a HiSeq 2500 platform, and more than 30–40 million raw paired-end reads were generated for each sample. For data analysis, read pairs in FASTQ format (50 bp/50 bp) were assessed for quality using FastQC (v.0.11.5) and mapped to the human genome hg19 with STAR2.5.2b using standard settings for paired reads. Uniquely mapped reads were assigned to annotated genes with HTSeq (v.0.6.1p1) with default settings. Read counts were normalized by library size, and differential gene expression analysis based on a negative binomial distribution was performed using DESeq2 (v.3.4). Gene set enrichment analysis was performed using previously curated gene sets.

### scRNA-seq data collection

At the time of sample collection, single-cell suspensions from each TGFβ treatment condition were collected and incubated with anti-human Hashtag antibodies conjugated to unique DNA barcodes (listed in Supplementary Table 5) and 1:100 human TruStain FcX (Fc Receptor Blocking Solution) (BioLegend, 422301) to prevent nonspecific binding. After three PBS washes, cells from different treatment conditions were pooled for single-cell droplet encapsulation and sequencing. The original sample sources of individual cells were then identified based on sequencing reads of the distinct Hashtag sequences. Two experimental replicates (samples 1 and 2) were collected and processed for scRNA-seq on consecutive days, following the same protocol described above. The two samples showed strong agreement, confirming their reproducibility as replicates, and were therefore combined for downstream analysis.

### scRNA-seq data analysis

#### Pre-processing of scRNA-seq data

FASTQ files of sequenced samples were processed using the SEQC pipeline (v.0.2.1) with default parameters for the 10× Genomics Single Cell 3’ library and mapped to the hg38 reference human genome. The SEQC pipeline performed read alignment, multi-mapping read resolution and cell barcode and unique molecular identifier (UMI) correction to generate a raw UMI-based (cell × gene) count matrix. An in-house method called SHARP (v.0.2.1; https://github.com/hisplan/sharp) subsequently assigned a cell as labeled by a particular Hashtag oligonucleotide, identifying the source of the cell as the TGFβ treatment condition barcoded with that oligonucleotide or as a doublet or low-quality droplet for removal. Following SHARP, we used the Scanpy software (v.1.6.0) in Python (v.3.8.5) to evaluate multiple complementary quality control metrics and further select high-quality single-cell droplets using the following steps. (1) To eliminate residual empty droplets, we filtered out cells with a library size (that is, total number of UMIs) below the saddle point of the size distribution histogram, corresponding to approximately the 5th percentile value. (2) To exclude apoptotic cells, we filtered out droplets with a high fraction of mitochondrial gene transcripts (mtRNA% ≥ 0.2), indicative of cellular stress. (3) To clean out any remaining lower-quality droplets, we used Scanpy’s pp.normalize_total function to normalize the count matrices by library size, performed principal component analysis and ran PhenoGraph (v.1.5.6; *k* = 30) to cluster the droplets. We examined the gene–gene expression covariance of each PhenoGraph cluster computed on its top 500 highly expressed genes and removed cells constituting the PhenoGraph clusters that did not exhibit apparent covariance structures. (4) After the above filtering steps, we ran DoubletDetection (v.2.5.2; https://github.com/dpeerlab/DoubletDetection) to infer and remove any potential droplets that may still contain more than one cell. The yield after pre-processing steps is listed in Supplementary Table 6.

#### Basic analysis of single-cell transcriptome

Following pre-processing of scRNA-seq data, we normalized the filtered count matrix by the library size computed on biologically relevant and robustly detected genes. We log-transformed the normalized count matrix (base = 2, pseudo count = 1) and performed principal component analysis using the top 5,000 highly variable genes. The top principal components, determined according to the knee point of total variance explained (122 principal components, 0.26 variance), were used to construct a *k*-nearest neighbor graph (n_neighbors = 15) to generate a force-directed layout of the cells. A consistent number of principal components was also selected to compute diffusion components using the Palantir algorithm (v.1.1), which runs diffusion maps with an adaptive anisotropic kernel.

#### Computing gene signature scores

We used the score_genes function of Scanpy to compute signature scores on the original log-transformed, normalized expression for genes associated with quiescence, S phase, G2/M phase, cell cycling (combined S phase and G2/M phase genes) and EMT^69^. The gene signature score was calculated as the average expression of a set of genes, subtracted from the average expression of a reference set of genes that were randomly sampled to match the expression distribution of the given gene set. All gene sets used are listed in Supplementary Table 2.

#### Visualizing gene expression trends along TGFβ treatment

We used Palantir to evaluate the trends of TGFβ treatment status, relevant gene signature scores and *GSN*, *FN1*, *ITGB3* and *ITGAV* expression along the DC1 values of all cells, which represent their primary axis of phenotypic variation. Palantir applies a generalized additive model to derive a robust estimate of the nonlinear expression trends and estimate the standard error of prediction. As a convenient way of implementing the trend fit, we ran the compute_gene_trends function in Palantir, providing DC1 values instead of the default pseudo-time as the input of the function. The generalized additive model-predicted expression trends were visualized with a Seaborn heatmap in Python (v.3.8.5), with each feature standardized between 0 and 1 across all cells.

### Quantitative PCR with reverse transcription analysis

RNA was extracted from cells using the RNeasy Mini Kit (Qiagen, 74106). cDNA was generated using the Transcriptor First Strand cDNA Synthesis Kit (Roche, 04379012001). Relative gene expression was determined using Taqman assays (Life Technologies) or SYBR green assays (Life Technologies). Quantitative PCR was performed on the ViiA 7 Real-Time PCR System (Life Technologies). Housekeeping gene *GAPDH* was used as the internal control for calculating relative gene expression. Three technical replicates were performed and plotted for each experiment. All experiments were performed at least twice, with representative results shown. Primers used for qPCR analysis are listed in Supplementary Table 3.

### ChIP–PCR analysis

For ChIP, cells were crosslinked with 1% formaldehyde (Millipore Sigma, F8775) for 10 min and quenched with 0.125 M glycine for 5 min at room temperature. ChIP was performed using an assay kit (Millipore Sigma, 17-295) according to the manufacturer’s instructions. Antibodies against H3K27Ac (Active Motif, 39133; 5 µg per sample), H3K27me3 (Millipore Sigma, 07-449; 10 µg per sample) and H3K4me1 (Abcam, ab8895; 5 µg per sample) were used. Immunoprecipitated DNA was purified using the QIAquick PCR Purification Kit (Qiagen), followed by qPCR analysis. The amplification product was calculated as the percentage of the input, then normalized to the control experiment for each condition. ChIP–PCR primers are listed in Supplementary Table 3.

### Western immunoblotting and ELISA

Cells were lysed using RIPA cell lysis buffer (Cell Signaling Technology, 9806S) supplemented with a protease inhibitor cocktail (Roche, cOmplete, mini, EDTA-free protease inhibitor tablets, 11836170001) and a phosphatase inhibitor cocktail (Thermo Fisher Scientific, Halt Phosphatase Inhibitor Cocktail, 78427; 1:100). Protein concentration was measured using the Pierce BCA Protein Assay Kit (Thermo Fisher Scientific, 23227). Lysates were loaded in NuPAGE Novex 4–12% Bis-Tris gels (Thermo Fisher Scientific, NP0336BOX) and transferred to nitrocellulose membranes. Membranes were blocked with Odyssey blocking buffer (LI-COR Biosciences, 927-60001) and incubated overnight at 4 °C with primary antibodies diluted in blocking buffer. After washing with PBS containing 0.1% Tween three times, membranes were incubated with IRDye 680RD goat anti-mouse (LI-COR Biosciences, 926-68070; 1:10,000), IRDye 680RD goat anti-rat (LI-COR Biosciences, 926-68076; 1:10,000) or IRDye 800CW goat anti-rabbit (LI-COR Biosciences, 926-32211; 1:10,000) secondary antibodies. Signal was detected using an Odyssey CLx imager (LI-COR Biosciences). Antibodies used for immunoblotting are listed in Supplementary Table 2.

For determination of DKK1 protein levels, 2 × 10^4^ cells were plated in a six-well plate, followed by the indicated treatment. Fresh medium with SB-505124 or TGFβ was changed 24 h before supernatant collection. DKK1 levels were measured using the Human Dkk-1 Quantikine ELISA Kit (R&D Systems, DKK100B), following the manufacturer’s instructions. Cell numbers were determined at the time of supernatant collection for normalization.

### AFM

Cells were seeded on glass-bottom Petri dishes (FluoroDish FD35) and kept in cultured medium during the acquisition of stiffness maps. AFM images were captured with a Nanowizard V (JPK-Bruker) in QITM advanced mode (stiffness mapping) at 37 °C. For cell stiffness mapping, a 1 µm diameter spherical AFM probe (silicon nitride cantilever, nominal spring constant *k* = 0.2 N m^−1^; Bruker, SAA-SPH-1UM) was used. The spring constant of each AFM probe was measured by the thermal noise method. A total of 15–20 cells from each experimental group were measured in each session. Bright-field images of each cell were collected during AFM measurements in an inverted optical objective integrated with the AFM (Zeiss AxioObserver Z1). For the stiffness mapping, 1 nN setpoint was used (60 µm × 60 µm or 120 µm × 120 µm image size with 32 × 32 pixel resolution) to ensure 2–3 µm sample indentation. The data were processed with JPK Data Processing software using the Hertz model with a 0.5 Poisson ratio as a fit parameter. Stiffness histograms were obtained by identifying the stiffness values belonging to each cell (and not the substrate values, shown in black on force maps) through a mask, and plotting the results from each cell sample as a single population. All measurements made <2 µm above the substrate were excluded. Stiffness distribution histograms were obtained using the histogram analysis tool in Excel (Microsoft) after normalizing for the total number of data points.

### Flow cytometry

Following the described treatment, cells were trypsinized, washed and blocked with TruStain FcX PLUS (anti-mouse CD16/32) (BioLegend, 156603) or Human TruStain FcX (Fc Receptor Blocking Solution) (BioLegend, 422301) for 10 min on ice. Cells were then stained with antibodies in PBS supplemented with 2% FBS (PBS-2%F) for 30 min on ice. Cells were washed with PBS and resuspended in PBS-2%F. Fluorescence was detected using a 5-laser Aurora (Cytek Biosciences). Antibody information is listed in Supplementary Table 2.

### Lymphocyte cytotoxicity, degranulation and cytokine production assays

To isolate murine NK cells, splenocyte suspensions were prepared by mechanical dissociation. NK cells were purified by magnetic depletion of non-NK cells using NK Isolation Kit II (Miltenyi Biotec, 130-096-892) and separation with magnetic columns (Miltenyi Biotec, 130-042-401). NK cells were cultured in NK cell medium (RPMI 1640 medium supplemented with 10% FBS, β2-mercaptoethanol, non-essential amino acids, 10 mM HEPES, 0.5 mM sodium pyruvate, 2 mM L-glutamine and 10 IU ml^−1^ of penicillin–streptomycin) containing 1,000 U ml^−1^ of murine recombinant IL-2 (R&D Systems, 402-ML-020).

To isolate human NK cells, peripheral blood was collected from healthy donors using protocols approved by the MSKCC Institutional Review Board (nos. 06-107 and 95-054). The samples were processed under Biospecimen Research Protocol Institutional Review Board no. 16-1564. Donors provided informed written consent. Peripheral blood mononuclear cells were isolated by Ficoll gradient purification. NK cells were purified using the NK Cell Isolation Kit (Miltenyi Biotec, 130-092-657) and expanded with C9 feeder cells at a 1:1 ratio in R10 media supplemented with 200 IU ml^−1^ human recombinant IL-2 (PeproTech, 200-02).

For measuring NK killing of cancer cells, target cancer cells plated in three or four replicates in 96-well plates were labeled with CellTracker Green CMFDA Dye (Thermo Fisher Scientific, C7025) and incubated with NK cells at a 1:4 carcinoma-to-NK cell ratio for 4 h at 37 °C. Cell mixtures were stained with 7-AAD Viability Staining Solution (Thermo Fisher Scientific, 00-6993-50) or DAPI to assess cancer cell cytolysis by flow cytometry.

For CTL cytotoxicity assays, cancer cells plated in triplicate in 96-well plates were loaded with varying concentrations of OVA peptide (1 nM, 0.3 nM, 0.01 nM, 0.03 nM, 0 nM) for 2 h and washed three times in culture medium. To assess killing, OT1 CTLs were added at a 4:1 effector-to-target ratio and incubated for 5 h at 37 °C in culture medium. Cells were then labeled with allophycocyanin-conjugated anti-CD8a antibody (Tonbo Biosciences, 20-0081), and specific lysis of target cells (GFP^+^CD8^−^ cells) was determined based on incorporation of either propidium iodide (Thermo Fisher Scientific, P3566) or DAPI (Invitrogen, D1306) using flow cytometry. All in vitro cytotoxicity assays were performed at least twice, with representative results shown.

To assess lytic granule secretion, a 2:1 CTL-to-cancer cell ratio was used. Cells were incubated for 90 min at 37 °C in the presence of eFluor660-conjugated anti-Lamp1 antibody (1 μg ml^−1^; Clone 1D4B, eBiosciences). Cells were then labeled with anti-CD8a antibody, and the percentage of CTLs (CD8^+^ cells) with positive Lamp1 staining was quantified by flow cytometry. To assess cytokine production, 4:1 CTL-to-cancer cell admixtures were incubated for 4 h at 37 °C in the presence of BD GolgiPlug protein transport inhibitor (BD Biosciences). Cells were then labeled with anti-CD8a antibody, fixed and permeabilized using the BD Cytofix/Cytoperm kit. After labeling with FITC-conjugated anti-TNF-α (BioLegend, 506304) and PE/Cy7-conjugated anti-IFNγ (BioLegend, 505826) antibodies, the percentage of CTLs (CD8^+^) expressing TNF and IFNγ was determined by flow cytometry. All assays were performed in three or four technical replicates.

### Cell motility assay

Time-lapse microscopy was performed on an inverted microscope (Zeiss AxioObserver Z1) using a ×10/0.45 NA objective. A total of 2,000 H2087-LCC cells were seeded in a six-well plate and treated with TGFβ for 3 days or 7 days before imaging. The temperature was set to 37 °C in the incubation chamber (incubator XLmulti S1). Cells at six different positions were imaged every 30 min for 24 h. Both phase contrast images with 100 ms exposure time and fluorescent images (GFP; excitation, 488 nm; emission, 509 nm) with 20 ms exposure time were acquired. Excitation was performed using an LED lamp (Colibri 7). The images were recorded using a digital camera (Hamamatsu, c4742-98) at 0.645 μm per pixel resolution using 1,345 pixel × 1,025 pixel image sizes at 12-bit depth. The software for image acquisition was Zen (v.2.6).

Image pre-processing (contrast adjusting, background subtraction, segmentation) was performed using Fiji^70^. Centroids of cells were identified by our customized MATLAB script, and tracks were generated using a previously released MATLAB implementation of IDL tracking methods (https://site.physics.georgetown.edu/matlab/code.html). All tracks were reviewed by cross-comparison with centroid movies and removed manually when they were invalid. Cell types were categorized at the start into more rounded (type 1) and less rounded (type 0) types according to a threshold value of 0.6 in roundness.

For each cell track, mean squared displacement (MSD) was calculated over a range of lag times as previously described^71^. The log–log plot of MSD versus lag time provides information about both the diffusion coefficient (intercept) and persistence (slope, α; reflecting motility) of cells. For a cell moving randomly, α = 1; for a cell with an applied force, α > 1; and for a cell that is constrained, α < 1; α was quantified as the slope using the fitlme function in MATLAB with the model log(MSD) ~ 1 + cellType × log(τ) (ref. ^72^). The model fits the first quarter of log(MSD) points obtained from each trajectory, as MSDs present large statistical fluctuations when τ is large^71^.

### Statistics and reproducibility

No statistical methods were used to predetermine sample sizes. Appropriate statistical tests were used to analyze data, as described in each figure legend. Data distribution was assumed to be normal, but this was not formally tested. Statistical analyses were performed with GraphPad Prism (v.10) software. The number of samples (*n*) is indicated in each figure panel or figure legend. For xenograft studies, female mice were randomly allocated into different groups before starting the treatments. All in vitro experiments were repeated at least twice, with representative results presented in the manuscript. No data were excluded from any analyses. The Investigators were not blinded to allocation during experiments and outcome assessment.

### Reporting summary

Further information on research design is available in the Nature Portfolio Reporting Summary linked to this article.

## Supplementary information

Reporting Summary

Supplementary Video 1 **Morphological change of H2087-LCC cells in the presence of TGF-β, related to** **Fig. 2**. A time-lapse movie of H2087-LCC cell pre-treated with TGF-β for 5 days and imaged in the presence of TGF-β for 36 hours. Time in HH:MM is indicated in the upper left corner. Scale bar: 50 μm.

Supplementary Video 2 **NK cell-mediated killing of TGF-β-treated H2087-LCC cell, related to** **Fig. 6**. A time-lapse movie of NK cells attacking a round and an elongated H2087-LCC cells pretreated with TGF-β for 5 days in the presence of Propidium Iodide (PI). Target cell death is associated with structural collapse and PI influx (red fluorescence). Time in HH:MM is indicated in the upper left corner. Scale bar: 50 μm.

Supplementary Table 1–6 Supplementary Tables 1–6.

## Source data

Source Data Fig. 1 Statistical Source Data.

Source Data Fig. 2 Statistical Source Data.

Source Data Fig. 2 Unprocessed western blots.

Source Data Fig. 3 Statistical Source Data.

Source Data Fig. 4 Statistical Source Data.

Source Data Fig. 4 Unprocessed western blots.

Source Data Fig. 5 Statistical Source Data.

Source Data Fig. 6 Statistical Source Data.

Source Data Fig. 7 Statistical Source Data.

Source Data Extended Data Fig. 1 Statistical Source Data.

Source Data Extended Data Fig. 1 Unprocessed western blots.

Source Data Extended Data Fig. 2 Statistical Source Data.

Source Data Extended Data Fig. 2 Unprocessed western blots.

Source Data Extended Data Fig. 3 Statistical Source Data.

Source Data Extended Data Fig. 4 Statistical Source Data.

Source Data Extended Data Fig. 5 Statistical Source Data.

Source Data Extended Data Fig. 6 Statistical Source Data.

Source Data Extended Data Fig. 6 Unprocessed western blots.

Source Data Extended Data Fig. 7 Statistical Source Data.

Source Data Extended Data Fig. 8 Statistical Source Data.

## Data Availability

Raw sequencing reads and processed files for RNA-seq have been deposited in the Gene Expression Omnibus database (GEO) under the SuperSeries accession number GSE269762. Raw sequencing reads and processed files for scRNA-seq have been deposited in GEO under SuperSeries accession number GSE295578. All data are publicly available as of the date of publication. All software programs used for analyses are publicly available and described in the Methods. Source data are provided with this paper.

## Code availability

No code was developed for this study.
