# Supplementary material for: TGFβ induces an atypical EMT to evade immune mechanosurveillance in lung adenocarcinoma dormant metastasis
Source: Nat Cancer. 2026 Jan 5;7(1):131–49. doi: 10.1038/s43018-025-01094-y (PMC12858408; doi:10.1038/s43018-025-01094-y)
Supplement: Supplementary file 1 — Reporting Summary [file 43018_2025_1094_MOESM1_ESM.pdf]

nature portfolio

Corresponding author(s): Joan Massagué

Last updated by author(s): 08/25/2025

Reporting Summary

Nature Portfolio wishes to improve the reproducibility of the work that we publish. This form provides structure for consistency and transparency in reporting. For further information on Nature Portfolio policies, see our Editorial Policies and the Editorial Policy Checklist.

Statistics

For all statistical analyses, confirm that the following items are present in the figure legend, table legend, main text, or Methods section.

n/a	Confirmed	
☐	☒	The exact sample size (*n*) for each experimental group/condition, given as a discrete number and unit of measurement
☐	☒	A statement on whether measurements were taken from distinct samples or whether the same sample was measured repeatedly
☐	☒	The statistical test(s) used AND whether they are one- or two-sided<br>*Only common tests should be described solely by name; describe more complex techniques in the Methods section.*
☒	☐	A description of all covariates tested
☐	☒	A description of any assumptions or corrections, such as tests of normality and adjustment for multiple comparisons
☐	☒	A full description of the statistical parameters including central tendency (e.g. means) or other basic estimates (e.g. regression coefficient) AND variation (e.g. standard deviation) or associated estimates of uncertainty (e.g. confidence intervals)
☐	☒	For null hypothesis testing, the test statistic (e.g. *F*, *t*, *r*) with confidence intervals, effect sizes, degrees of freedom and *P* value noted<br>*Give P values as exact values whenever suitable.*
☒	☐	For Bayesian analysis, information on the choice of priors and Markov chain Monte Carlo settings
☒	☐	For hierarchical and complex designs, identification of the appropriate level for tests and full reporting of outcomes
☒	☐	Estimates of effect sizes (e.g. Cohen's *d*, Pearson's *r*), indicating how they were calculated

Our web collection on statistics for biologists contains articles on many of the points above.

Software and code

Policy information about availability of computer code

Data collection	Bioluminescence (BLI) data was acquired using IVIS Spectrum Xenogen Instrument (PerkinElmer). Immunofluorenscence imaging data was acquired with a Zeiss Axio Imager Z1 microscope (Carl Zeiss) or an SP5 confocal microscope (Leica Microsystems). Time-lapse microscopy was performed on an inverted microscope (Zeiss AxioObserver Z1) using a 10x/0.45 NA objective. qRT-PCR data was acquired using ViiA 7 real-time PCR system (LIfe Technology). RNA sequencing samples were quantified by Ribogreen and quality assessed by Agilent BioAnalyzer. Multiplexed sequencing libraries were run on a Hiseq2500 platform. Western blot data was acquired using Odyssey CLx imager (LI-COR Biosciences). Flow cytometry analysis was acquired using LSRFortessa (BD Biosciences). ELISA data was collected using Synergy H1 Hybrid microplate reader (Agilent). AFM images were captured with an Nanowizard V (JPK-Bruker) in QITMadvanced Mode (stiffness mapping).
Data analysis	Statistical analysis: GraphPad Prism (v10.3.0)<br>Bioluminescence (BLI) data analysis: LivingnImage software (v 4.8.0).<br>Fluorescent image processing and analysis: ImageJ software (v2.14.0).<br>Flow cytometry data analysis: FlowJo (v10.0.0)<br>qRT-PCR analysis: QuantStudio (v1.5)<br>RNA-sequencing analysis: FastQC (v0.11.5), STAR (v2.5.2), HTSeq (v0.6.1p1), DESeq2 (v3.4), Gene set enrichment analysis (GSEA) was performed using previously curated gene sets.<br>Western blot images and analysis: ImageStudioLite (v5.2.5)<br>ELISA analysis: Gen5 (v2.09)

For manuscripts utilizing custom algorithms or software that are central to the research but not yet described in published literature, software must be made available to editors and reviewers. We strongly encourage code deposition in a community repository (e.g. GitHub). See the Nature Portfolio guidelines for submitting code & software for further information.

Data

Policy information about availability of data

All manuscripts must include a data availability statement. This statement should provide the following information, where applicable:

- Accession codes, unique identifiers, or web links for publicly available datasets
- A description of any restrictions on data availability
- For clinical datasets or third party data, please ensure that the statement adheres to our policy

Raw sequencing reads and processed files for RNA-seq have been deposited in the Gene Expression Omnibus database (GEO) under the SuperSeries accession number GEO: GSE269762. Raw sequencing reads and processed files for scRNA-seq have been deposited under the SuperSeries accession number GEO: GSE295578 and will be publicly available as of the date of publication. Reviewer token for GSE295578 is: ghsdosogttuftap. ChIP-seq data were from Hu et al (GSE210946). No custom computer code was generated for this study. Codes for conducting the scRNA-seq analysis will be available upon request.

Research involving human participants, their data, or biological material

Policy information about studies with human participants or human data. See also policy information about sex, gender (identity/presentation), and sexual orientation and race, ethnicity and racism.

Reporting on sex and gender	N/A
Reporting on race, ethnicity, or other socially relevant groupings	N/A
Population characteristics	N/A
Recruitment	To isolate human NK cells, peripheral blood was collected from healthy donors using protocols approved by the Memorial Sloan Kettering Cancer Center Institutional Review Board (nos. 06-107 and 95-054). The samples were processed under Biospecimen Research Protocol Institutional Review Board no. 16-1564. Donors provided informed written consent.
Ethics oversight	To isolate human NK cells, peripheral blood was collected from healthy donors using protocols approved by the Memorial Sloan Kettering Cancer Center Institutional Review Board (nos. 06-107 and 95-054). The samples were processed under Biospecimen Research Protocol Institutional Review Board no. 16-1564. Donors provided informed written consent.

Note that full information on the approval of the study protocol must also be provided in the manuscript.

Field-specific reporting

Please select the one below that is the best fit for your research. If you are not sure, read the appropriate sections before making your selection.

☒ Life sciences ☐ Behavioural & social sciences ☐ Ecological, evolutionary & environmental sciences

For a reference copy of the document with all sections, see nature.com/documents/nr-reporting-summary-flat.pdf

Life sciences study design

All studies must disclose on these points even when the disclosure is negative.

Sample size	For in vivo experiments, no statistical method was used to predetermine the sample size. Sample sizes were chosen based on prior experience ad pilot experiment for detecting statistically significant differences between conditions. In compliance with IACUC guidelines, a minimal number of animals for a statistically significant result was used.
Data exclusions	No data was excluded from the studies.
Replication	All attempts at replication were successful. Biological replicates of each experiment is stated under each figure legend and all attempts were successful. The key findings were verified independently by multiple models.
Randomization	For in vivo treatments with immune cell depleting antibodies, mice were distributed into treatment groups with approximately equal bioluminescent intensities. For all other experiments, samples were randomly assigned to each group.
Blinding	Investigators were not blinded to treatment groups for the in vivo and in vitro studies, as knowledge of this information was essential to conduct the studies.

Reporting for specific materials, systems and methods

We require information from authors about some types of materials, experimental systems and methods used in many studies. Here, indicate whether each material, system or method listed is relevant to your study. If you are not sure if a list item applies to your research, read the appropriate section before selecting a response.

Materials & experimental systems

n/a	Involved in the study
☐	☒ Antibodies
☐	☒ Eukaryotic cell lines
☒	☐ Palaeontology and archaeology
☐	☒ Animals and other organisms
☒	☐ Clinical data
☒	☐ Dual use research of concern
☒	☐ Plants

Methods

n/a	Involved in the study
☒	☐ ChIP-seq
☐	☒ Flow cytometry
☒	☐ MRI-based neuroimaging

Antibodies

Antibodies used

anti-mouse IgG2a control, Immune depletion in mouse, Bio X Cell, Cat# BE0089, clone 2A3, 200ug/mouse;
anti-mouse NK1.1 Immune depletion in mouse, Bio X Cell, Cat# BE0036, clone PK136, 200ug/mouse;
anti-mouse CD4 Immune depletion in mouse, Bio X Cell, Cat#BE0003-1 , clone GK1.5, 200ug/mouse;
anti-mouse CD8a Immune depletion in mouse, Bio X Cell, Cat# BE0004-1, clone 53-6.7, 200ug/mouse;
rabbit anti-asialo-GM1, Immune depletion in mouse, Wako Chemical Cat#986-10001, 33ug/mouse;
anti-mouse CD45, Flow cytometry, Tonbo Biosciences, Cat# 35-0041-U100, 30-F11, violetFluor 450, 1:200;
anti-mouse CD4, Flow cytometry, Tonbo Biosciences, Cat# 35-0041-U100, GK1.5, FITC, 1:200;
anti-mouse CD8a, Flow cytometry, Tonbo Biosciences, Cat# 60-0081-U100, 53-6.7, PE-Cy7, 1:200;
anti-mouse CD8a, Flow cytometry, Tonbo Biosciences, Cat# 20-0081-U100, 53-6.7, APC, 1:200;
anti-mouse NK1.1, Flow cytometry, Tonbo Biosciences, Cat# 20-5941-U100, PK136, APC, 1:200;
anti-mouse Lamp1, Flow cytometry, eBiosciences, Cat# 50-112-8662, 1D4B, eFluor660, 1ug/ml;
anti-mouse TNF-a, Flow cytometry, BioLegend, Cat# 506304, FITC, 1ug/ml;
anti-mouse IFN-g, Flow cytometry, BioLegend, Cat# 505826, PE/Cy7, 1ug/ml;
anti-mouse H-2Kb/H-2Db, Flow cytometry, BioLegend, Cat#114617, APC/Fire™ 750; 1:1000
anti-human HLA-A,B,C Antibody, Flow cytometry, BioLegend, Cat##311409, APC; 1:1000
Rat anti-Ki67, IF, Invitrogen, Cat# 14-5698-80, 1/1000;
Mouse mAb anti-Vimentin, IF,Abcam Cat# ab8069, 1/500;
Chicken pAb anti-GFP, IF, Aves Labs Cat# GFP-1010 1/250;
Rat anti-CD31, IF, BD Biosciences, Cat# 550274, 1/250;
Rabbit pAb anti-mCherry, IF, Abcam, Cat# ab167453, 1/500;
Chicken pAb anti-mCherry, IF, Abcam, Cat# ab205402, 1/500;
Rat mAb anti-E-cadherin, IF, Abcam, Cat# ab11512, 1/200;
Rabbit pAb anti-Fibronectin, IF, Abcam, Cat# ab2413, 1/250;
Rabbit mAb anti-beta catenin, IF, Abcam, Cat# ab32572, 1/500;
Rabbit pAb anti-ZO1, IF, Abcam, Cat# ab96587, 1/500;
Mouse mAb anti-EpCAM, IF, Cell Signaling Technology, Cat# 2929S, 1/200;
Cytokeratin Pan Antibody, IF, Thermo Fisher Scientific, Cat# MA5-13203, 1/100;
Mouse mAb anti-p27, IF, Cell Signaling Technology, Cat# 3698, 1/100;
Alexa Fluor 568 Phalloidin, IF, Thermo Fisher Scientific, Cat# A12380, 5 unit/mL;
Alexa Fluor 647 Phalloidin, IF, Thermo Fisher Scientific, Cat# A22287, 5 unit/mL;
goat anti-chicken 488, IF, Thermo Fisher Scientific, Cat# A-11039, 1/500;
goat anti-mouse 488, IF, Thermo Fisher Scientific, Cat# A-21121, 1/500;
goat anti-chicken 594, IF, Thermo Fisher Scientific, Cat# A-11042, 1/500;
goat anti-rabbit 594, IF, Thermo Fisher Scientific, Cat# A-11012, 1/500;
goat anti-rat 594, IF, Thermo Fisher Scientific, Cat# A-11007, 1/500;
goat anti-rat 647, IF, Thermo Fisher Scientific, Cat# A-21247, 1/500;
goat anti-rabbit 647, IF, Thermo Fisher Scientific Cat# A-21245, 1/500;
Rat mAb anti-Sox2, Western blot, Thermo Fisher Scientific, Cat# 14-9811-82, 1/200;
Rabbit mAb anti-Sox9, Western blot, Abcam, Cat# ab185966, 1/1000;
Rabbit mAb anti-Sox9, Western blot, Cell Signaling Technology, Cat# 82630S, 1/1000;
Rabbit mAb anti-NKX2-1, Western blot, Abcam, Cat# ab76013, 1/1000;
Rabbit mAb anti-NKX2-1, Western blot, Cell Signaling Technology, Cat# 12373S, 1/1000;
Mouse mAb anti-TGFBR2, Western blot, Santa Cruz, Cat# sc-17792, 1/100;
Rabbit mAb anti-E-cadherin, Western blot, Cell Signaling Technology, Cat# 3195, 1/500;
Rabbit mAb anti-beta catenin, Western blot, Abcam, Cat# ab32572, 1/1000;
Rabbit mAb anti-integrinb3, Western blot, Cell Signaling Technology, Cat# 13166, 1/1000;
Rabbit mAb anti-Vimentin, Western blot, Cell Signaling Technology, Cat# 5741, 1/1000;
Rabbit pAb anti-Fibronectin, Western blot, Abcam, Cat# ab2413, 1/500;
Rabbit pAb anti-gelsolin, Western blot, Thermo Fisher Scientific, Cat# 11644-2-AP, 1/1000;
Mouse mAb anti-MYLK2, Western blot, Santa Cruz, Cat# sc-58803, 1/500;
Mouse mAb anti-beta-actin, Western blot, Cell Signaling Technology, Cat# 3700, 1/5000;
Rabbit pAb anti-beta-actin, Western blot, Cell Signaling Technology, Cat# 4967S, 1/3000;
Goat anti-mouse IRDye 680RD, LI-COR Biosciences, Cat# 926-68070, 1:10000;
Goat anti-rat IRDye 680RD, LI-COR Biosciences, Cat# 926-68076, 1:10000;

Goat anti-rabbit IRDye 800CW, LI-COR Biosciences, Cat# 926-32211, 1:10000;
Rabbit anti-H3K27Ac, Active Motif, Cat# 39133, 5 µg per sample;

Validation

Validation statement for each primary antibody is provided on the manufacturer's website.
anti-mouse CD45: https://cytekbio.com/products/violetfluor-450-anti-mouse-cd45-30-f11?variant=40581181734948;
anti-mouse CD4:https://cytekbio.com/products/fitc-anti-mouse-cd4-gk1-5?variant=40581224038436;
anti-mouse CD8a: https://cytekbio.com/products/pe-cyanine7-anti-mouse-cd8a-53-6-7?variant=40581200871460;
anti-mouse CD8a: https://cytekbio.com/products/apc-anti-mouse-cd8a-53-6-7?variant=40581236555812;
anti-mouse NK1.1: https://cytekbio.com/products/pe-cyanine7-anti-mouse-nk1-1-cd161-pk136?variant=40581200412708;
anti-mouse Lamp1: https://www.fishersci.com/shop/products/cd107a-lamp-1-monoclonal-antibody-ebio1d4b-1d4b-efluor-660-ebioscience-invitrogen/501128662;
anti-mouse TNF-a: https://www.biolegend.com/ja-jp/products/fitc-anti-mouse-tnf-alpha-antibody-976?GroupID=GROUP24;
anti-mouse IFN-g: https://www.biolegend.com/fr-ch/products/pe-cyanine7-anti-mouse-ifn-gamma-antibody-5865?GroupID=GROUP24;
anti-mouse H-2Kb/H-2Db: https://www.biolegend.com/en-us/products/apc-fire-750-anti-mouse-h-2kb-h-2db-antibody-16326;
anti-human HLA-A,B,C Antibody: https://www.biolegend.com/en-us/products/apc-anti-human-hla-a-b-c-antibody-1870;
Rat anti-Ki67:https://www.thermofisher.com/antibody/product/Ki-67-Antibody-clone-SolA15-Monoclonal/14-5698-82;
Mouse mAb anti-Vimentin:https://www.abcam.com/en-us/products/primary-antibodies/vimentin-antibody-v9-cytoskeleton-marker-ab8069;
Chicken pAb anti-GFP:https://www.antibodiesinc.com/products/epitope-tag-and-gfp-antibodies/green-fluorescent-protein-gfp-antibody;
Rat anti-CD31: https://www.bdbiosciences.com/en-us/products/reagents/flow-cytometry-reagents/research-reagents/single-color-antibodies-ruo/purified-rat-anti-mouse-cd31.550274;
Rabbit pAb anti-mCherry: https://www.abcam.com/en-us/products/primary-antibodies/mcherry-antibody-ab167453;
Chicken pAb anti-mCherry: https://www.abcam.com/en-us/products/primary-antibodies/mcherry-antibody-ab205402;
Rat mAb anti-E-cadherin:https://www.abcam.com/en-us/products/primary-antibodies/e-cadherin-antibody-decma-1-intercellular-junction-marker-ab11512;
Rabbit pAb anti-Fibronectin: https://www.abcam.com/en-us/products/primary-antibodies/fibronectin-antibody-ab2413;
Rabbit mAb anti-beta catenin:https://www.abcam.com/en-us/products/primary-antibodies/beta-catenin-antibody-e247-chip-grade-ab32572;
Rabbit pAb anti-ZO1: https://www.abcam.com/en-us/products/primary-antibodies/zo1-tight-junction-protein-antibody-ab96587;
Mouse mAb anti-EpCAM: https://www.cellsignal.com/products/primary-antibodies/epcam-vu1d9-mouse-mab/2929;
Cytokeratin Pan Antibody: https://www.thermofisher.com/antibody/product/Cytokeratin-Pan-Antibody-clone-PAN-CK-Cocktail/MA5-13203;
Mouse mAb anti-p27: https://www.cellsignal.com/products/primary-antibodies/p27-kip1-sx53g8-5-mouse-mab/3698?Ntt=3698S&_=1533637173549&tahead=true;
Alexa Fluor 568 Phalloidin: https://www.thermofisher.com/order/catalog/product/A12380;
Alexa Fluor 647 Phalloidin: https://www.thermofisher.com/order/catalog/product/A22287;
Rat mAb anti-Sox2: https://www.thermofisher.com/antibody/product/SOX2-Antibody-clone-Btjce-Monoclonal/14-9811-82;
Rabbit mAb anti-Sox9: https://www.abcam.com/en-us/products/primary-antibodies/sox9-antibody-epr14335-78-ab185966;
Rabbit mAb Sox9:https://www.cellsignal.com/products/primary-antibodies/sox9-d8g8h-rabbit-mab/82630?srsltid=AfmBOopVAyv4g6Ns7HWbiVGQ2tiSbrEY6p5qAT55gSnwEVseZFbO0Gwe;
Rabbit mAb anti-NKX2-1: https://www.abcam.com/en-us/products/primary-antibodies/ttf1-nkx2-1-antibody-ep1584y-ab76013;
Rabbit mAb anti-NKX2-1: https://www.cellsignal.com/products/primary-antibodies/thyroid-transcription-factor-1-ttf-1-d2e8-rabbit-mab/12373?srsltid=AfmBOookfUs1wBpL55DOoJA5PNp_PrPa2JWOspfsQhef0ezQVS_M3sXR;
Mouse mAb anti-TGFBR2: https://www.scbt.com/p/tgfbeta-rii-antibody-e-6;
Rabbit mAb anti-E-cadherin: https://www.cellsignal.com/products/primary-antibodies/e-cadherin-24e10-rabbit-mab/3195;
Rabbit mAb anti-beta catenin: https://www.abcam.com/en-us/products/primary-antibodies/beta-catenin-antibody-e247-chip-grade-ab32572;
Rabbit mAb anti-integrin b3: https://www.cellsignal.com/products/primary-antibodies/integrin-b3-d7x3p-xp-rabbit-mab/13166?Ntt=13166S&_=1533633893696&tahead=true;
Rabbit mAb anti-Vimentin: https://www.cellsignal.com/products/primary-antibodies/vimentin-d21h3-xp-rabbit-mab/5741;
Rabbit pAb anti-gelsolin: https://www.thermofisher.com/antibody/product/Gelsolin-Antibody-Polyclonal/11644-2-AP;
Mouse mAb anti-MYLK2: https://www.scbt.com/p/mylk2-antibody-my-21;
Mouse mAb anti-beta-actin: https://www.cellsignal.com/products/primary-antibodies/b-actin-8h10d10-mouse-mab/3700;
Rabbit pAb anti-beta-actin: https://www.cellsignal.com/products/primary-antibodies/b-actin-antibody/4967;
Rabbit anti-H3K27Ac: https://www.activemotif.com/catalog/details/39133/histone-h3-acetyl-lys27-antibody-pab;

Eukaryotic cell lines

Policy information about cell lines and Sex and Gender in Research

Cell line source(s)

H2087 (ATCC)-LCC were isolated as described in Malladi et al. 2016, Cell. H2087-SO cells were derived from spontaneous metastases in mice inoculated intracardially with H2087-LCC cells. Mouse lung cancer cell line 802T4 and 393T3 were a gift from T. Jacks (Kock Institute MIT). A549 cells were purchased from ATCC.

Authentication

H2087-LCC and A549 were authenticated with STR profiling. 802T4 cell was genotyped to verify the presence of KrasG12D and Tp53 mutation using PCR amplification.

Mycoplasma contamination

All cell lines tested negative for mycoplasma contamination.

Commonly misidentified lines
(See ICLAC register)

None of the cell lines used are listed as commonly misidentified lines in the ICLAC database.

Animals and other research organisms

Policy information about studies involving animals; ARRIVE guidelines recommended for reporting animal research, and Sex and Gender in Research

Laboratory animals	All animal experiments were performed in accordance with protocols approved by the Memorial Sloan Kettering Cancer Center Institutional Animal Care and Use Committee (IACUC) (protocol 99-09-032). Maximum tumor burden was not exceeded in the animal experiments. Athymic nude mice were obtained from Envigo (strain #: 069) or Charles River Laboratories (strain #: 490). NSG (NOD.Cg-PrkdcscidIL2rgtm1Wjl/SzJ, strain #005557), B6129SF1/J (strain #101043) and B6(Cg)-Tyrc-2J/J (B6-albino, strain #000058) mouse strains were obtained from the Jackson Laboratory. Female mice 6 to 8 weeks of age were used for in vivo studies. 2 to 6 months-old male and female OT1 αβTCR transgenic mice (Jackson Laboratories, Strain #:003831) were used to generate OT1 CTLs for in vitro assays. All animals were housed under specific pathogen-free conditions.
Wild animals	This study does not involve wild animals.
Reporting on sex	Sex was not considered in the design of this study.
Field-collected samples	This study does not involve samples collected in the field.
Ethics oversight	All animal experiments were performed in accordance with protocols approved by the Memorial Sloan Kettering Cancer Center Institutional Animal Care and Use Committee (IACUC).

Note that full information on the approval of the study protocol must also be provided in the manuscript.

Plants

Seed stocks	N/A
Novel plant genotypes	N/A
Authentication	N/A

Flow Cytometry

Plots

Confirm that:

- [x] The axis labels state the marker and fluorochrome used (e.g. CD4-FITC).
- [x] The axis scales are clearly visible. Include numbers along axes only for bottom left plot of group (a 'group' is an analysis of identical markers).
- [x] All plots are contour plots with outliers or pseudocolor plots.
- [x] A numerical value for number of cells or percentage (with statistics) is provided.

Methodology

Sample preparation	For validating the depletion of immune cells, peripheral blood (75 microliters) was collected into 1ml PBS containing 5 mM EDTA (10 microliters of 0.5 M stock) and mix immediately to prevent clotting. Keep tubes on ice. Remove RBCs from samples. Centrifuge cells for 5 min at 500 × g and resuspend with 1ml RBC lysis buffer. Incubate at room temperature for 10mins. Cells should be washed 2-3x with FACS buffer (PBS supplemented with either 1% BSA or 5% FBS and containing 0.05% NaN 3). Incubate cells in FcBlock CD16/32 (1:100 in PBS) for 15mins on ice. Centrifuge cells for 5 min at 500 × g. Suspend the pellet from the final wash in 50 microliters FACS buffer per each analysis on a single sample - up to three separate staining reactions can be set up from a single sample). Add 50 microliters of cell suspension to 10 microliters of antibody solution and mix gently. Incubate for 30 minutes on ice. Wash cells 2-3x with FACS buffer and suspend in 200-300 microliters FACS buffer for analysis.
Instrument	Flow cytometric analysis on an LSRFortessa (BD Biosciences) instrument.
Software	Flow cytometry data was analyzed with FlowJo (v10.0.0)
Cell population abundance	No cell sorting was involved in this study.

Gating strategy

Leukocytes were gated as CD45+ cell. NK cells were gated as CD45+,NK1.1+. T cells were gated as CD45+,CD4+ T cells or CD45+,CD8+ T cells. Peripheral blood from NSG mice was used as a negative control.

☐ Tick this box to confirm that a figure exemplifying the gating strategy is provided in the Supplementary Information.
