# Supplementary figures and images for: TGFβ induces an atypical EMT to evade immune mechanosurveillance in lung adenocarcinoma dormant metastasis
Source: Nat Cancer. 2026 Jan 5;7(1):131–49. doi: 10.1038/s43018-025-01094-y (PMC12858408; doi:10.1038/s43018-025-01094-y)

Source Data: Figure 2g

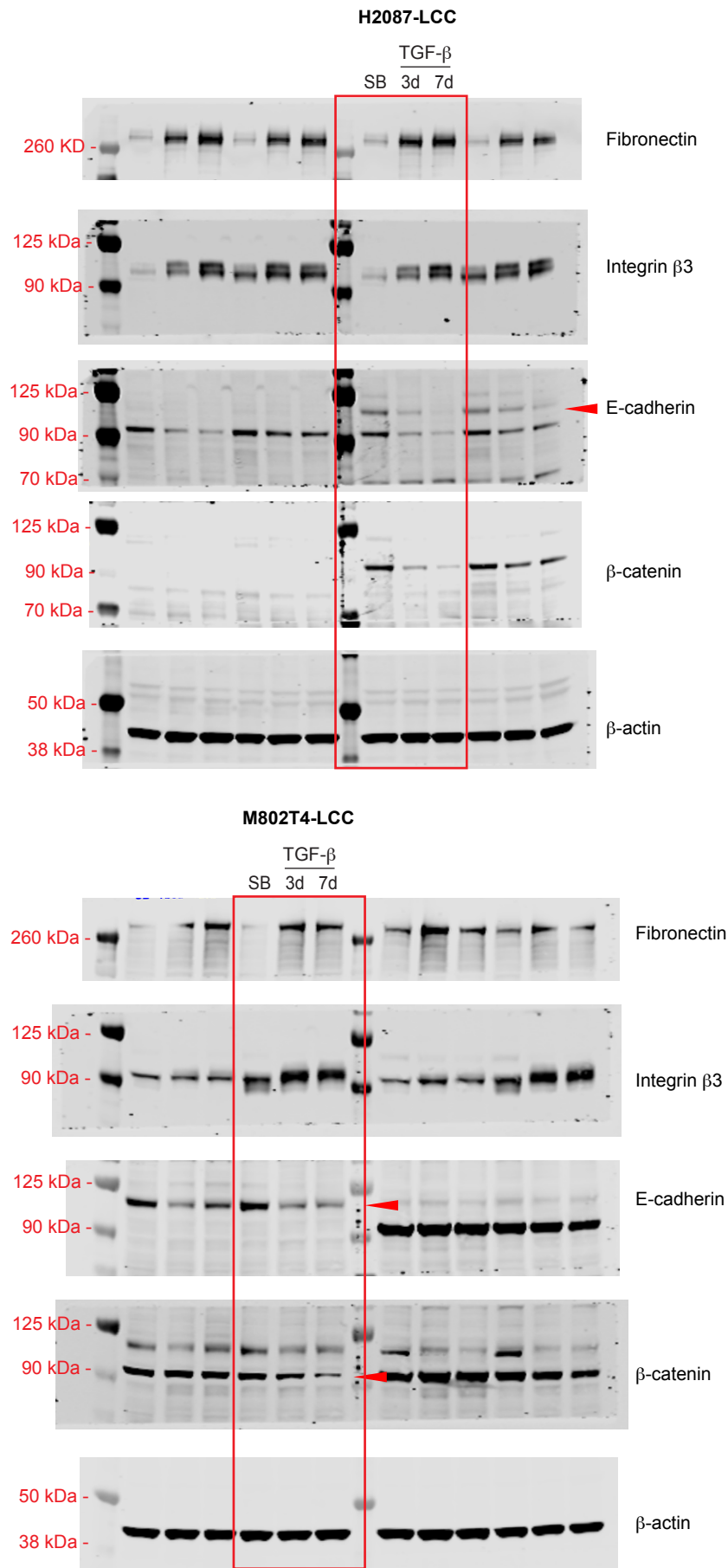

Supplement: Supplementary file 7 — Unprocessed western blots. [file 43018_2025_1094_MOESM7_ESM.pdf]

Source Data: Figure 4

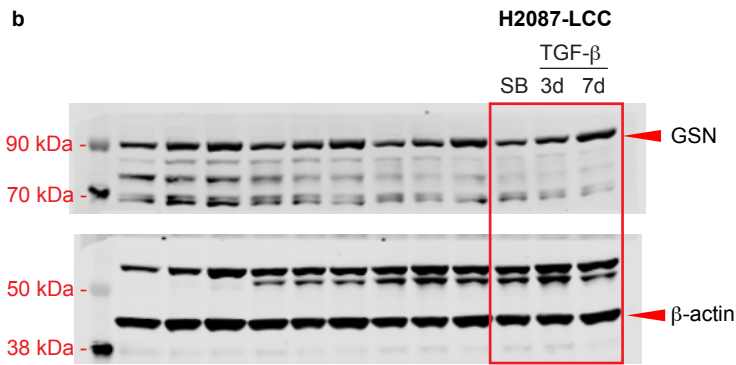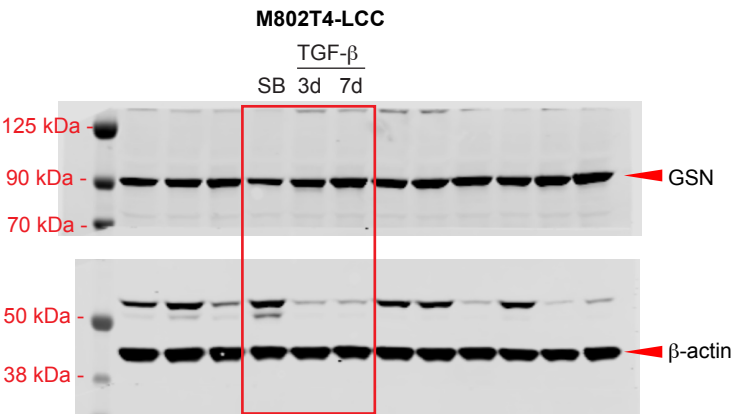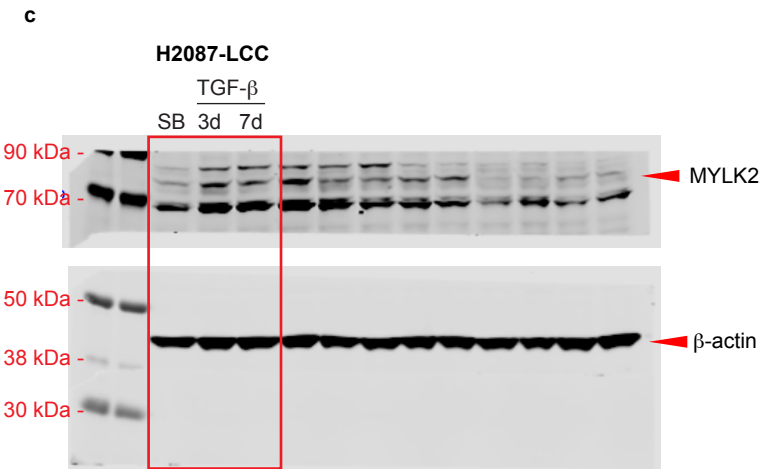

Supplement: Supplementary file 10 — Unprocessed western blots. [file 43018_2025_1094_MOESM10_ESM.pdf]

Source Data: ED Figure 1k

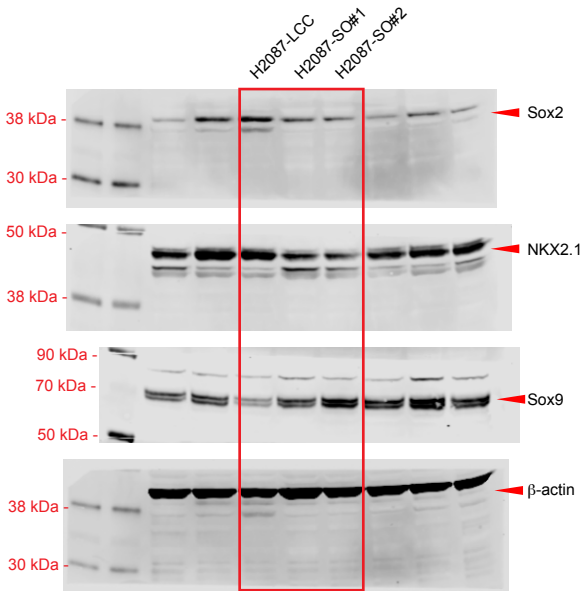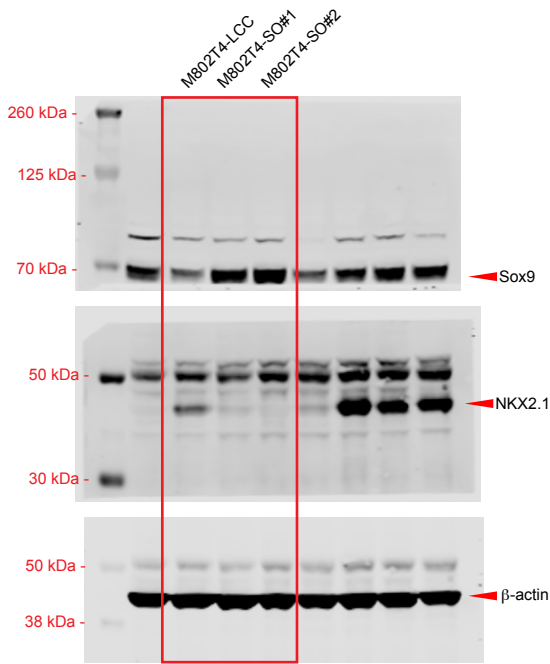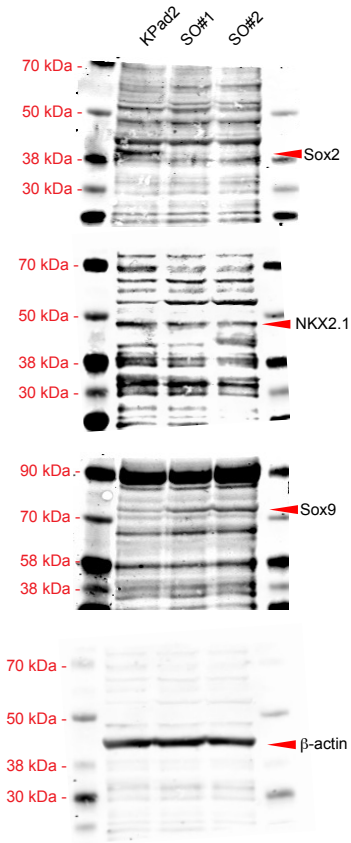

Supplement: Supplementary file 15 — Unprocessed western blots. [file 43018_2025_1094_MOESM15_ESM.pdf]

**Source Data: ED Figure 2**

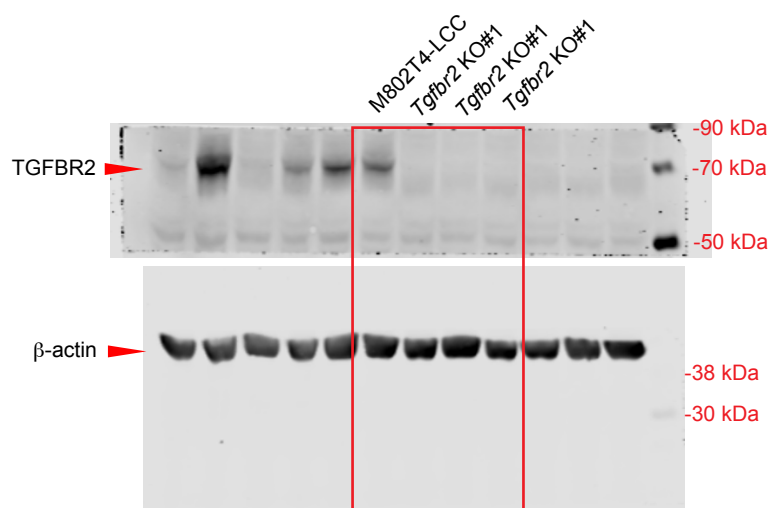

Supplement: Supplementary file 17 — Unprocessed western blots. [file 43018_2025_1094_MOESM17_ESM.pdf]

Source Data: ED Figure 6b

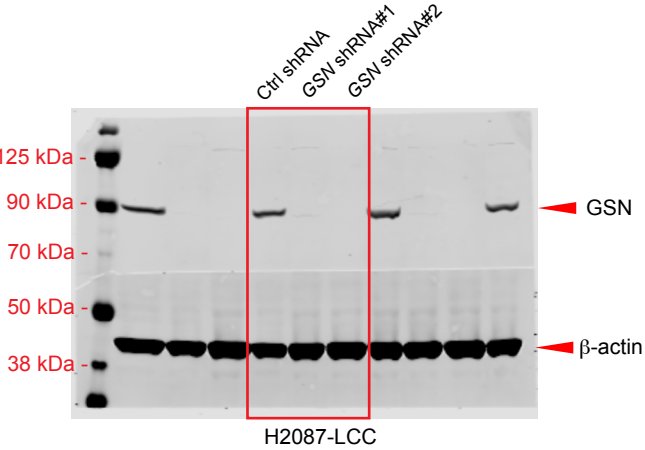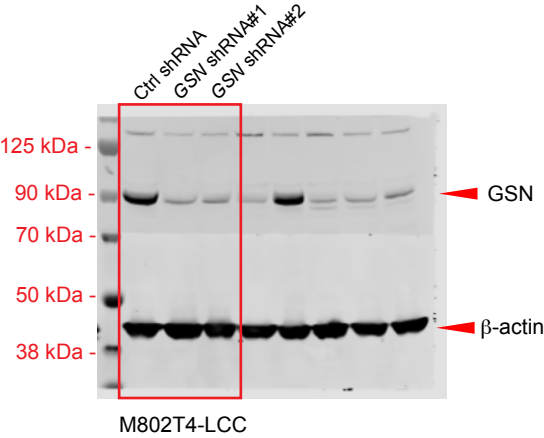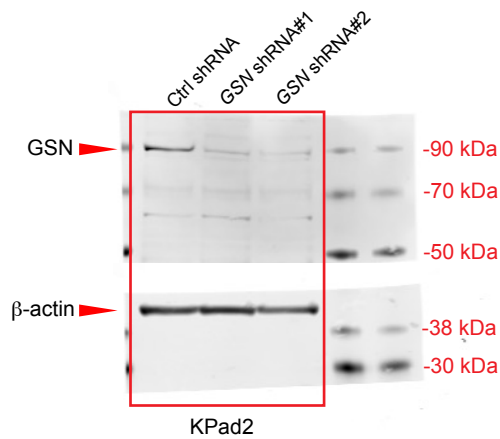

Supplement: Supplementary file 22 — Unprocessed western blots. [file 43018_2025_1094_MOESM22_ESM.pdf]
